# The E3 ubiquitin ligase Itch is required for B-cell development

**DOI:** 10.1038/s41598-018-36844-9

**Published:** 2019-01-23

**Authors:** Xiaoling Liu, Yu Zhang, Yinxiang Wei, Zhiding Wang, Gaizhi Zhu, Ying Fang, Bing Zhai, Ruonan Xu, Gencheng Han, Guojiang Chen, He Xiao, Chunmei Hou, Beifen Shen, Yan Li, Ning Ma, Renxi Wang

**Affiliations:** 1 Laboratory of Immunology, Institute of Basic Medical Sciences, Beijing, 100850 China; 2 Department of Rheumatology, First hospital of Jilin University, Changchun, 130021 China; 3 Department of Nephrology, The 307th Hospital of Chinese People’s Liberation Army, Beijing, 100850 China; 4 College of Pharmacy, Henan University, Kaifeng, 475001 China; 5 Laboratory of Cellular and Molecular Immunology, Henan University, Kaifeng, Henan 475001 China; 6 Department of Geriatric Hematology, Nanlou Division, Chinese PLA General Hospital; National Clinical Research Center for Geriatric Diseases, Beijing, 100853 China; 7 College of Life Science and Technology, Xinjiang University, Urumqi, Xinjiang 830046 China

## Abstract

The E3 ubiquitin ligase Itch interacts with Foxo1 and targets it for ubiquitination and degradation during follicular helper T-cell differentiation, whereas the transcription factor Foxo1 plays a critical role in B-cell development. Thus, we proposed that Itch mediates B-cell differentiation. Unexpectedly, we found that Itch deficiency downregulated Foxo1 expression in B cells. Itch cKO (conditional knock out in B cells) mice had fewer pro-B cells in the bone marrow, more small resting IgM^−^IgD^−^B cells in the periphery, and lower B-cell numbers in the lymph nodes through decreased Foxo1-mediated IL-7Rα, RAG, and CD62L expression, respectively. Importantly, Itch deficiency reduced Foxo1 mRNA expression by up-regulating JunB-mediated miR-182. Finally, Foxo1 negatively regulated JunB expression by up-regulating Itch. Thus, we have identified a novel regulatory axis between Itch and Foxo1 in B cells, suggesting that Itch is essential for B-cell development.

## Introduction

B cells and their antibodies are the central elements of humoral immunity and, as part of the adaptive immune system, protect against a nearly unlimited variety of pathogens. Defects in B-cell development, selection, and function lead to autoimmunity, malignancy, immunodeficiencies, and allergy^1^. B-cell development begins in the bone marrow and continues in secondary lymphoid organs^2^. B cells develop from a lymphoid precursor in bone marrow that transits sequentially through the pro-B cell, pre-BI, large and small pre-BII, and immature B-cell stages^3^. Pro-B cells (CD43^+^B220^+^CD19^+^c-kit^+^) constitute the earliest progenitor group committed to the B-cell lineage^4^. Recombination-activating gene (Rag) proteins appear to be expressed at this stage, promoting Ig gene recombination, which is required for the process of B lymphopoiesis^5^. This rearrangement machinery is precisely regulated by several transcription factors, including PU.1, E2A, early B-cell factor (EBF) and Pax5^6,7^. Apart from transcription factors, lymphocyte development also requires cytokines that positively and negatively regulate gene expression. Marrow stromal cell–derived interleukin-7 (IL-7) is a nonredundant cytokine in murine B-cell development that promotes V-to-DJ rearrangements and transmits survival/proliferation signals^8^. A pro-B cell block in development can occur due to two primary types of defects: failed IL-7R signaling and failed pre-BCR assembly and signaling^9^.

Immature B cells leave the bone marrow, and travel through the blood to the spleen to complete maturation. The adhesion molecule L-selectin (CD62L) initiates the tethering and rolling of cells and allows subsequent transmigration from the bloodstream into tissues^10,11^. CD62L has a prominent role in controlling the recirculation and distribution of leukocyte subsets within non-inflamed and inflamed tissues^12^. Blocking antibodies against CD62L have been shown to inhibit lymphocyte binding to HEVs both *in vitro* and *in vivo*^13^, whereas CD62L knockout mice display a 70% to 90% reduction in lymph node cellularity^14^. These studies suggest that CD62L is the lymph node homing receptor^15^.

Foxo1 has been reported to regulate the development of early B-cell precursors, peripheral B-cell homeostasis, and terminal differentiation^16^. Foxo1 deficiency impairs pro-B and pre-B development and results in fewer lymph node B cells by downregulating IL-7R, RAG, and CD62L, respectively^9^. Specifically, decreased Rag expression and heavy chain gene rearrangement at the pro-B cell stage may result in small resting pre-B (IgM^−^IgD^−^) cells that transit to the periphery^9^. Increased expression of Foxo1 in combination with STAT5 activation resulting from IL7R signaling, has been suggested to activate the transcription of the Ebf1 gene, which encodes the transcription factor EBF1^17^. Taken together, Foxo1 is an important node in the dynamic network of transcription factors that orchestrate B-cell differentiation and specialization^16^.

The HECT-type E3 ubiquitin ligase Itch has been shown to interact with Foxo1 and targets it for ubiquitination and degradation^18^. Itch is absent in non-agouti-lethal 18 H or Itchy mice, which develop a severe immunological disease. Several Itch substrates are relevant to epidermal development and homeostasis, such as p63, Notch, c-Jun and JunB^19^. The role of Itch in T cells has been widely studied. Itch^−/−^ αβ and γδ T cells independently contribute to autoimmunity in Itchy mice^20^. Further, Itch is associated with and indces the ubiquitination of JunB, a transcription factor that is involved in Th2 differentiation^21^. In addition, Itch expression by Treg cells controls Th2 inflammatory responses through the ubiquitination of IL-4^22^. A recent study has shown that Itch is required for follicular helper T-cell (Tfh-cell) differentiation by associating with Foxo1 and promoting its ubiquitination and degradation.

It is unclear whether Itch affects B-cell development. In the present study, we found that Itch deficiency resulted in low Foxo1 expression in B cells. Consequently, Foxo1-regulated IL-7R, RAG and CD62L expression was decreased, lowering the population of pro-B cells and mature IgM^+^IgD^+^B cells, increasing the number of small resting IgM^−^IgD^−^B cells in the periphery, and reducing the B-cell count in LNs, repectively. Further, Itch deficiency downregulated Foxo1 mRNA by upmodulating JunB-mediated miR-182. Thus, we have found a novel regulatory axis in Itch-controlled Foxo1 expression in B cells suggesting that Itch is essential for B-cell development.

## Results

### B-cell differentiation requires Itch

To investigate the role of the E3 ubiquitin ligases Itch in B-cell differentiation, we first examined Itch expression in B cells. A previous study has suggested that Itch is expressed in splenic B cells^23^. We found splenic IgM^−^IgD^−^, IgM^+^IgD^−^, and IgM^+^IgD^+^ B cells (the gating strategy for sorting is described in Supplementary Figure 1a) and B220^+^CD19^+^ CD43^+^IgM^−^ pro B cells, B220^+^CD19^+^CD43^−^IgM^−^ pre-B cells, and B220^+^CD19^+^CD43^−^ IgM^+^IgD^−^ immature B cells in bone marrow (BM) express Itch protein (Supplementary Figures 2a,b).

To perform B-cell-specific depletion of Itch, mice with loxP sites inserted into the Itch gene (Itch^F/F^) were crossed with mice that express Cre-recombinase (Cre) under the control of the CD19 promoter (CD19^Cre^) (Supplementary Figure 3a). To confirm B-cell-specific depletion of Itch, we measured Itch expression in CD19^Cre^Itch^F/F^ (Itch conditional knocked out Itch cKO) mice and their WT littermates. The results demonstrated that Itch was knocked out in B-cells from the spleen and BM of Itch cKO mice (Supplementary Figure 2c). Critically, by co-IP assay, Itch bound to Foxo1 (Supplementary Figure 2d), which is required at multiple stages of B-cell differentiation^9^. These results suggest that Itch may play an important role in B-cell differentiation.

**Figure 1:**
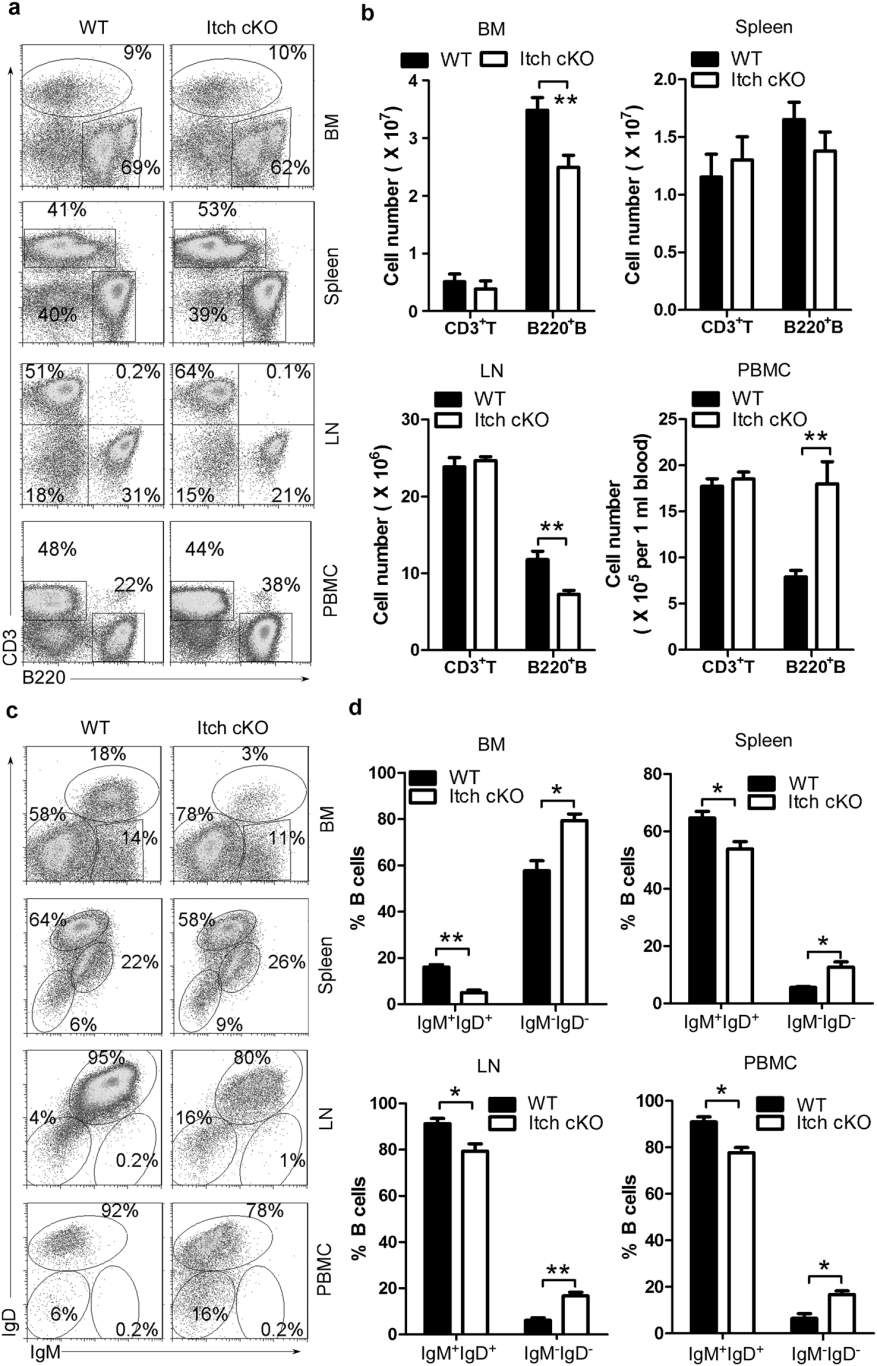
Itch is essential for B-cell development. Representative flow cytometry (FACS) profiles (**a**) and the absolute numbers (**b**) of CD3^+^B220^−^ and CD3^−^B220^+^ B cells and representative flow cytometry profiles (**c**) and the absolute numbers (**d**) of IgM^+^IgD^+^B cells and IgM^−^IgD^−^B-cells in gated CD3^−^B220^+^ B cells in bone marrow (BM), spleen, lymph nodes (LNs), and peripheral blood monocytes (PBMCs) from Itch cKO mice and WT littermates (n = 12 per group, 7~9 weeks old). (**b**,**d**) two-way ANOVA plus Bonferroni post-tests to compare each column with control column. Error bars, s.e.m. *P < 0.05, **P < 0.01.

To determine whether Itch affects B-cell development, we first analyzed CD3^−^B220^+^ B cells. We found that compared with their WT littermates, Itch cKO mice had more total B cells in peripheral blood monocytes (PBMCs) and fewer B cells in bone marrow (BM) and lymph nodes (LNs) (Fig. 1a,b). However, total B-cell numbers did not change significantly in the spleen of Itch cKO mice (Fig. 1a,b). In addition, T-cell numbers did not change significantly in the BM, spleen, LN and PBMCs in Itch cKO mice (Fig. 1a,b). Importantly, there were fewer mature IgM^+^IgD^+^B cells in the BM, PBMCs, spleen, and LNs, whereas there were more IgM^−^IgD^−^B cells in the periphery, such as inPBMCs, spleen, and LNs (Fig. 1c,d). Altogether, these data suggest that Itch may play an important role in B-cell differentiation.

### Itch cKO mice show similar abnormal B-cell development as Foxo1 cKO mice

**Figure 2:**
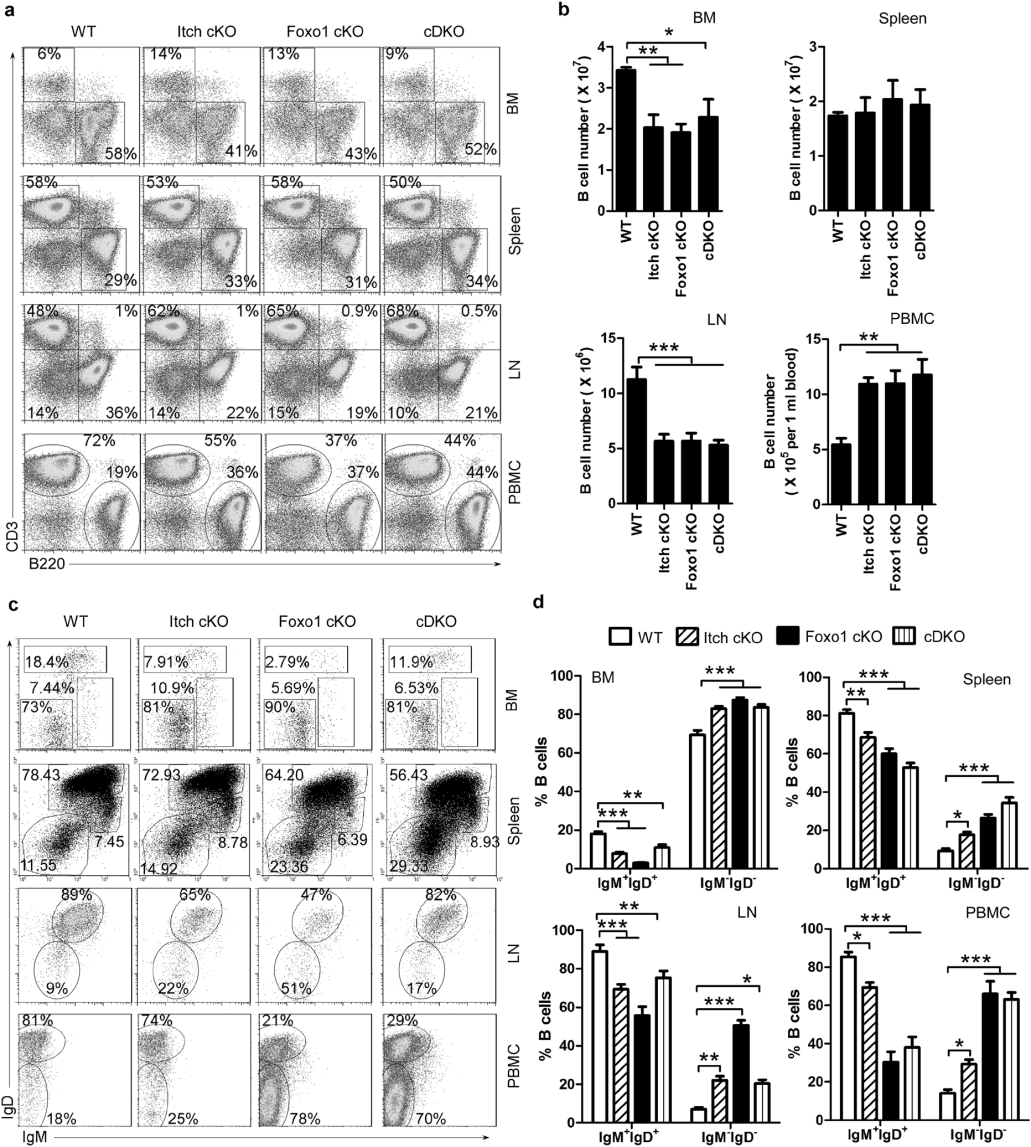
Similar abnormal B-cell development in both Itch cKO and Foxo1 cKO mice. Representative FACS profiles (**a**) and the absolute numbers (**b**) of CD3^+^B220^−^ and CD3^−^B220^+^ B cells and representative flow cytometry profiles (**c**) and the absolute numbers (**d**) of IgM^+^IgD^+^B cells and IgM^−^IgD^−^B-cells in gated CD3^−^B220^+^ B cells in BM, spleen, LNs, and PBMCs from CD19^cre^Itch^F/+^Foxo1^F/+^ (WT), CD19^cre^Itch^F/F^Foxo1^F/+^ (Itch cKO), CD19^cre^Itch^F/+^Foxo1^F/F^ (Foxo1 cKO), and CD19^cre^Itch^F/F^Foxo1^F/F^ mice (Itch-Foxo1 cDKO) (n = 20 mice per group, 7~9 weeks old). One-way (**b**) and two-way (**d**) ANOVA plus Bonferroni post-tests to compare each column with control column. Error bars, s.e.m. *P < 0.05, **P < 0.01, ***P < 0.001.

A previous study has shown that Itch deficiency blocks the differentiation of follicular helper T (Tfh) cells by up-regulating Foxo1 expression^18^. Thus, to explore the role of Foxo1 in the effect of Itch on B-cell differentiation, we developed double conditional knockout mice (Supplementary Figure 3c–e). We found that total B-cell numbers were increased in PBMCs, decreased in BM and LNs, and unchanged in the spleen in Itch cKO, Foxo1 cKO, and Itch-Foxo1 cDK mice (Fig. 2a,b, Supplementary Figure 4). In addition, we found fewer mature IgM^+^IgD^+^B cells whereas and more small resting pre-B cells (IgM^−^IgD^−^) in the BM, PBMCs, spleen and LNs from Itch cKO, Foxo1 cKO, and Itch-Foxo1 cDK mice (Fig. 2c,d). A previous study has shown that small resting pre-B cells (IgM^−^IgD^−^) transit to the periphery in Foxo1-deficient mice^9^. In line with this study, we found that whereas a high proportion of IgM^−^IgD^−^ wild-type B cells expressed intracellular κ or λ light chain, suggesting that they are class-switched B cells, few IgM^−^IgD^−^ B cells from Itch cKO, Foxo1 cKO, and Itch-Foxo1 cDK mice expressed intracellular κ or λ light chains but abundant amounts of intracellular μ-heavy chain, suggesting that they are small resting pre-B cells (Supplementary Figure 5). Altogether, these results suggest that Itch deficiency has a similar effect on B-cell development as Foxo1 deficiency.

### Itch deficiency blocks B-cell development by reducing Foxo1 expression

**Figure 3:**
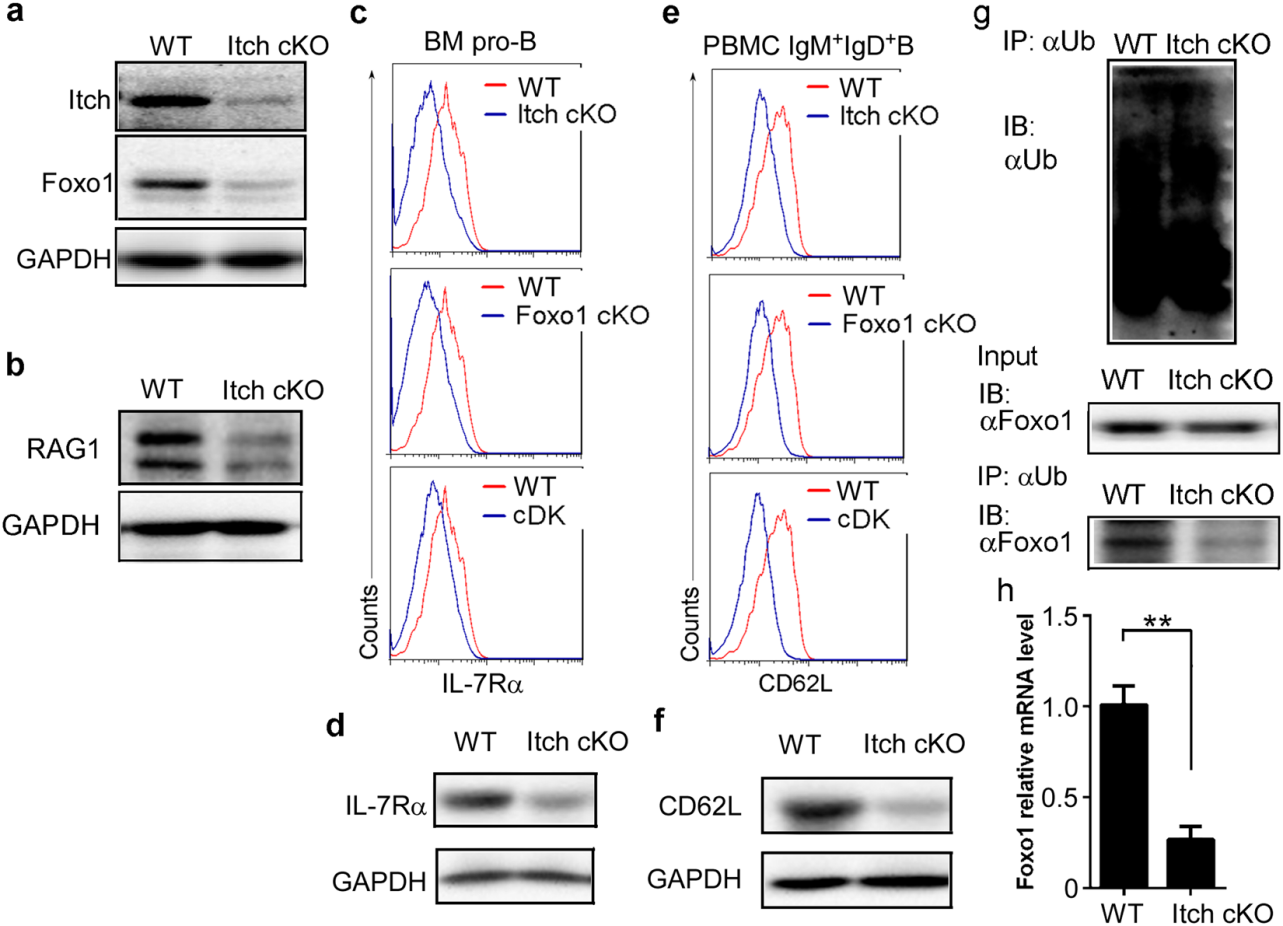
Itch deficiency blocks B-cell development by reducing Foxo1 expression. Immunoblot analysis of Itch and Foxo1 in splenic B cells (**a**), and RAG1 in pro-B cells (**b**) sorted from Itch cKO mice and WT littermates (n = 3 mice per group, 7~9 weeks old). Representative FACS profile indicating the expression of IL-7Rα in pro-B cells of BM (**c**) and CD62L in IgM^+^IgD^+^B cells (**e**) from WT, Itch cKO, Foxo1 cKO, and Itch and Foxo1 cDK mice (n = 4 mice per group, 7~9 weeks old). Immunoblot analysis of IL-7Rα expression in pro-B cells of BM (**d**) and CD62L expression in IgM^+^IgD^+^B cells of PBMC (**f**) sorted from Itch cKO mice and WT littermates (n = 3 mice per group, 7~9 weeks old). (**g**) Immunoassay of B220^+^ B cells sorted from 7–9 week-old Itch cKO mice and WT littermates (n = 3 mice per group, 7~9 weeks old), pretreated with MG132 and stimulated for 20 min with LPS (20 μg/ml), as assessed by denaturation of lysates in 1% SDS, immunoprecipitation with anti-ubiquitin (α-Ub) and immunoblot analysis with monoclonal anti-ubiquitin (P4D1; top blot) or anti-Foxo1 (bottom blot); lysates without immunoprecipitation (middle blot) probed with anti-Foxo1. (**h**) Real-time quantitative PCR (qPCR) analysis of Foxo1 mRNA in splenic B cells from WT and Itch cKO mice (n = 10 mice per group, 7~9 weeks old). Data are shown as fold-change relative to the expression in WT cells (arbitrarily set to 1), after normalization to GAPDH expression. (**a–h**) Results represent at least three independent experiments. (**h**) Two-tailed student’s t-test. Error bars, s.e.m. **P < 0.01.

Contrary to a previous study suggesting that Itch-deficient Tfh up-regulates Foxo1 expression^18^, we found that compared with CD19^cre^Itch^F/+^Foxo1^F/+^ mice, CD19^cre^Itch^F/F^Foxo1^F/+^ mice expressed less Foxo1 protein in splenic B cells (Supplementary Figure 3e). Furthermore, we found that Foxo1 protein was also reduced in CD19^cre^Itch^F/F^ mice (Fig. 3a). Critically, we observed that Itch deficiency also downregulated Foxo1-dependent gene expression, including RAG (Fig. 3b), IL-7Rα (Fig. 3c,d) and CD62L (Fig. 3e,f), which are critical in multiple stages of B-cell differentiation^9^. Overexpression of Foxo1 re-induced Rag1, IL-7Rα and CD62L expression in Itch^−/−^ pro-B, pro-B, and splenic B cells, respectively (Supplementary Figure 6), suggesting that Itch regulates B-cell differentiation via Foxo1.

To explore the mechanisms underlying Itch-reduced Foxo1 expression, we examined whether Itch could promoted Foxo1 ubiquitination and degradation. At similar levels of ubiquitin (Fig. 3g, top panel) and total Foxo1 protein (Fig. 3g, middle panel) in WT B cells and Itch^−/−^ B cells, Itch^−/−^ B cells had a lower level of Foxo1 ubiquitination than that in WT B cells (Fig. 3g, bottom panel). Thus, theoretically Itch deficiency should up-regulate Foxo1 protein. Therefore, we proposed that Itch deficiency reduces Foxo1 mRNA by an unknown mechanism. As expected, Foxo1 mRNA levels were decreased in Itch-deficient B cells (Fig. 3h).

### Itch deficiency reduces Foxo1 mRNA expression by up-regulating JunB expression

**Figure 4:**
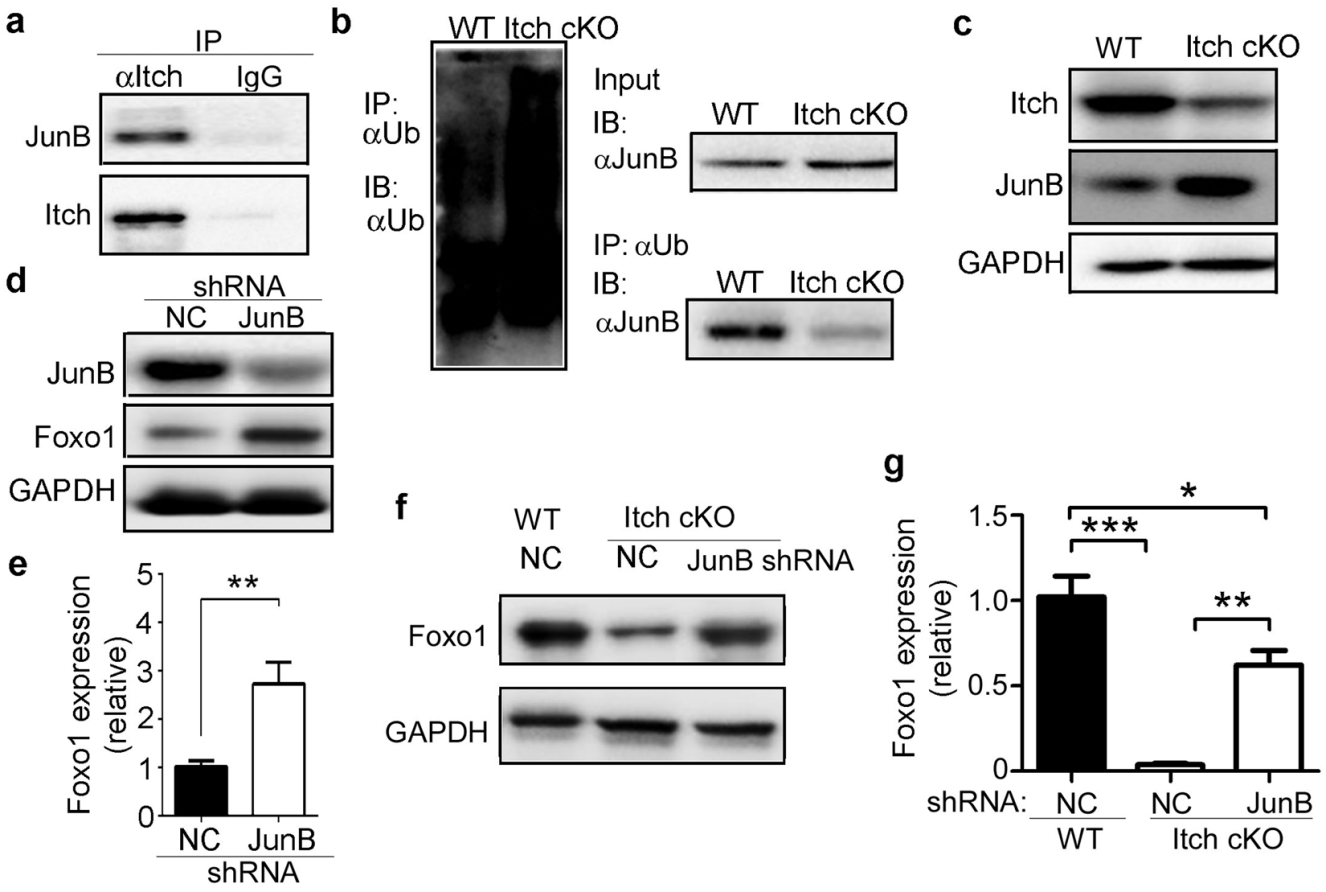
Itch deficiency reduces Foxo1 mRNA expression by up-regulating JunB expression. (**a**) Splenic B cells from 7~9-week-old C57BL/6 mice were subjected to immunoprecipitation with anti-Itch antibodies (αItch) and isotype control IgG and immunoblotting with antibodies that were reactive to the indicated proteins. (**b**) Immunoassay of B220^+^ B cells sorted from 7~9-week-old Itch cKO mice and WT littermates (n = 3 mice per group, 7~9 weeks old), pretreated with MG132 and stimulated for 20 min with LPS (20 μg/ml), as assessed by denaturation of lysates in 1% SDS, immunoprecipitation with anti-ubiquitin (α-Ub) and immunoblot analysis with monoclonal anti-ubiquitin (P4D1; left blot) or anti-JunB (right lower blot); lysates without immunoprecipitation (right top blot) probed with anti-JunB. (**c**) Immunoblot analysis of Itch and JunB in splenic B cells sorted from 7~9-week-old Itch cKO mice and WT littermates (n = 3 mice per group, 7~9 weeks old). (**d**,**e**) Splenic B cells sorted from 7~9-week-old C57BL/6 mice were infected with lentivirus containing negative control (NC) shRNA or JunB-specific shRNA and stimulated for 48 hours with LPS (1 μg/ml). The expression of JunB and Foxo1 protein (**d**), and Foxo1 mRNA (**e**) was analyzed by immunoblotting and qPCR, respectively. (**f**,**g**) Splenic B cells sorted from 7~9-week-old Itch cKO mice and WT littermates (n = 3 mice per group, 7~9 weeks old) were infected with lentivirus containing negative control (NC) shRNA or JunB-specific shRNA and stimulated for 48 hours with LPS (1 μg/ml). The expression of Foxo1 protein (**f**) and mRNA (**g**) was analyzed by immunoblotting and qPCR, respectively. (**e**,**g**) Relative mRNA levels are normalized to GAPDH mRNA expression and calculated relative to the mRNA expression in WT cells infected with NC shRNA, set to 1. (**a–g**) Results represent at least three independent experiments. Two-tailed student’s t-test (**e**) and one-way ANOVA plus Bonferroni post-tests (**g**) to compare each column with control column. Error bars, s.e.m. *P < 0.05, **P < 0.01, ***P < 0.001.

Itch is associated with and induces ubiquitination of JunB in type 2T helper (Th2) cell differentiation^21^ and JunB has been reported to reduce Foxo1 expression^24,25^. Thus, we proposed that Itch deficiency reduces Foxo1 mRNA expression by up-regulating JunB expression. As expected, we found that Itch could bind to JunB (Fig. 4a). Whereas the amount of ubiquitin (Fig. 4b, left panel) and total JunB protein (Fig. 4b, right top panel) in Itch^−/−^ B cells was slightly higher than in WT B cells, Itch^−/−^ B cells had a lower level of JunB ubiquitination than WT B cells (Fig. 4b, right lower panel). Accordingly, Itch-deficient B cells expressed a higher level of JunB (Fig. 4c). When JunB was depleted, the level of Foxo1 protein and mRNA increased in WT B cells (Fig. 4d,e) and Itch-deficient B cells (Fig. 4f,g). These results suggest that Itch deficiency reduces Foxo1 mRNA expression by up-regulating JunB expression.

### JunB promotes activation of the Foxo1 promoter

**Figure 5:**
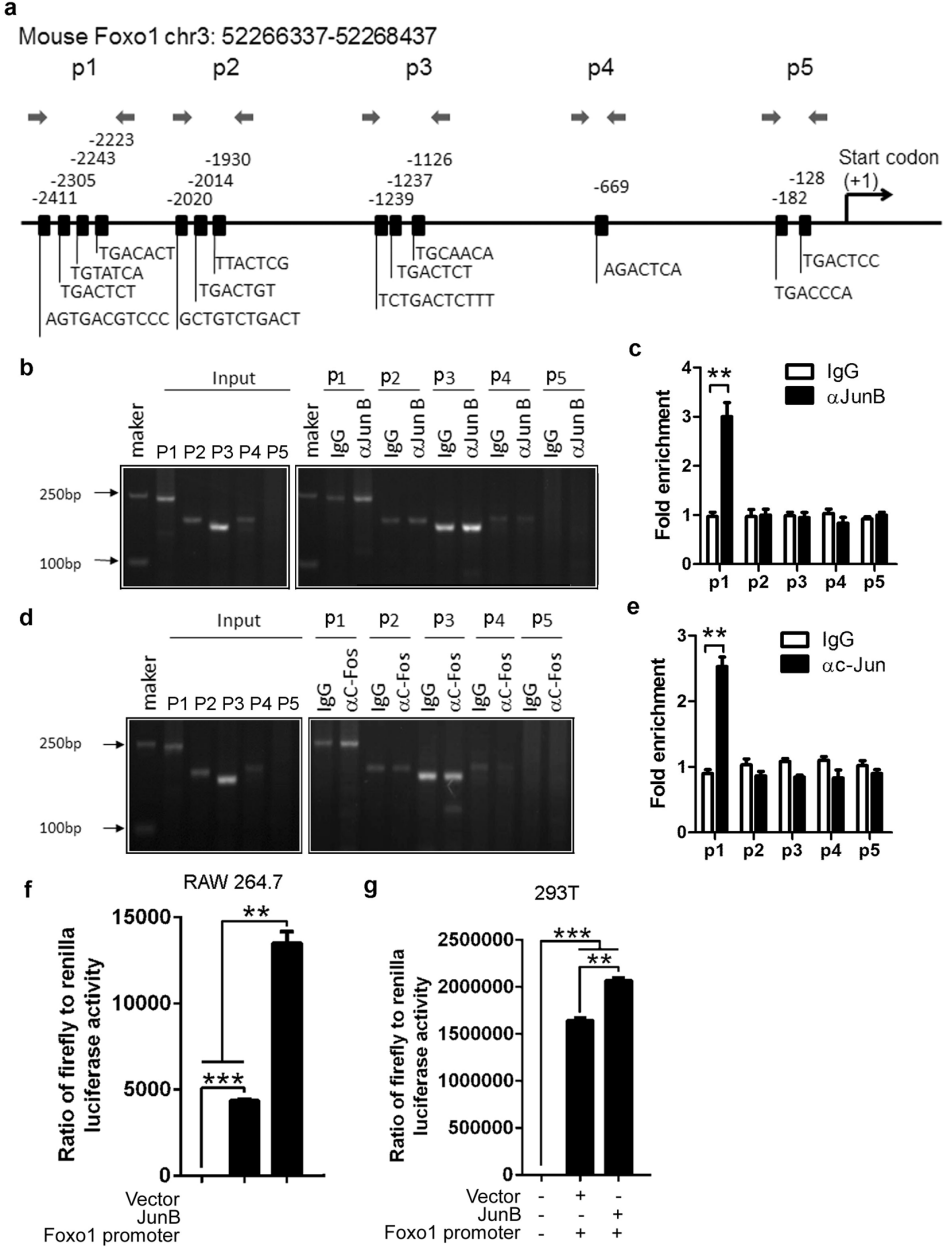
JunB promotes activation of the Foxo1 promoter. (**a**) Schematic diagram of mouse Foxo1 promoter region illustrating the positions of the primer pairs used for ChIP assays. Sequences represent the main predicted JunB binding sites from Supplementary Tables I and II. Arrows represent the region of the 5 primer pairs. ChIP assays of WT B220^+^ B cells using a JunB (αJunB) (**b**,**c**) and c-Fos (αc-Fos) (**d,e**) antibody or control IgG probing for the Foxo1 promoter locus. PCR (**b,d**) and qPCR (**c,d**) were used to analyze the enrichment and the fold-enrichments are representative of one of four independent experiments. (**c**,**e**) Data are shown as fold-change relative to IgG control (arbitrarily set to 1), after normalization to input. Empty vector Lv 201 (Vector) or Lv201/JunB (JunB) and luciferase reporter vector pEZX-PG04.1/Foxo1 promoter were co-transduced into RAW264.7 cells (**f**) or 293 T cells (**g**). Dual luciferase reporter gene expression was analyzed, and the results are shown as the ratio of firefly to Renilla luciferase activity. (**a–g**) The data represent at least three independent experiments. Two-tailed student’s t-test (**c**,**e**) and one-way ANOVA plus Bonferroni post-tests (**f**,**g**) to compare each column with control column. Error bars, s.e.m. **p < 0.01, ***p < 0.001.

As a transcription factor, JunB may bind the Foxo1 promoter to regulate Foxo1 mRNA expression. We chose and analyzed the Foxo1 promoter sequence (Supplementary Table I). By using web-based software, we found that the transcription factor JunB has binding sites in the Foxo1 promoter (Supplementary Table II). To prove whether JunB binds directly to the Foxo1 promoter, we used ChIP-PCR/qPCR technology. We first chose the main predicted binding sites of JunB to design the primer pairs for ChIP-PCR/qPCR (Fig. 5a). Anti-mouse JunB antibody or control IgG was used to probe the Foxo1 locus. The relative binding was defined by PCR and qPCR (Fig. 5b,c). The results suggested that JunB can bind the sites amplified by the p1 primer pairs (Fig. 5b,c). JunB is a subunit of AP-1, such as the JunB:c-Fos transcription factor complex^25^. Thus, we used anti-c-Fos antibody to further probe the JunB binding sites. As expected, like the anti-JunB antibody, anti-c-Fos antibody labeled the binding sites amplified by the p1 primer pairs (Fig. 5d,e). To explore the role of JunB in the activation of the Foxo1 promoter, we used a murine macrophage line RAW264.7, and 293T human embryonic kidney cells, which are suitable for inducing luciferase reporter vectors. Compared with the vector, JunB significantly increased the ratio of firefly to renilla luciferase activity (Fig. 5f,g). These data demonstrate that JunB directly promotes activation of the Foxo1 promoter. Thus, we proposed that JunB reduces Foxo1 mRNA on the post-transcriptional but not transcriptional level.

### JunB reduces Foxo1 mRNA by up-regulating miR-182

**Figure 6:**
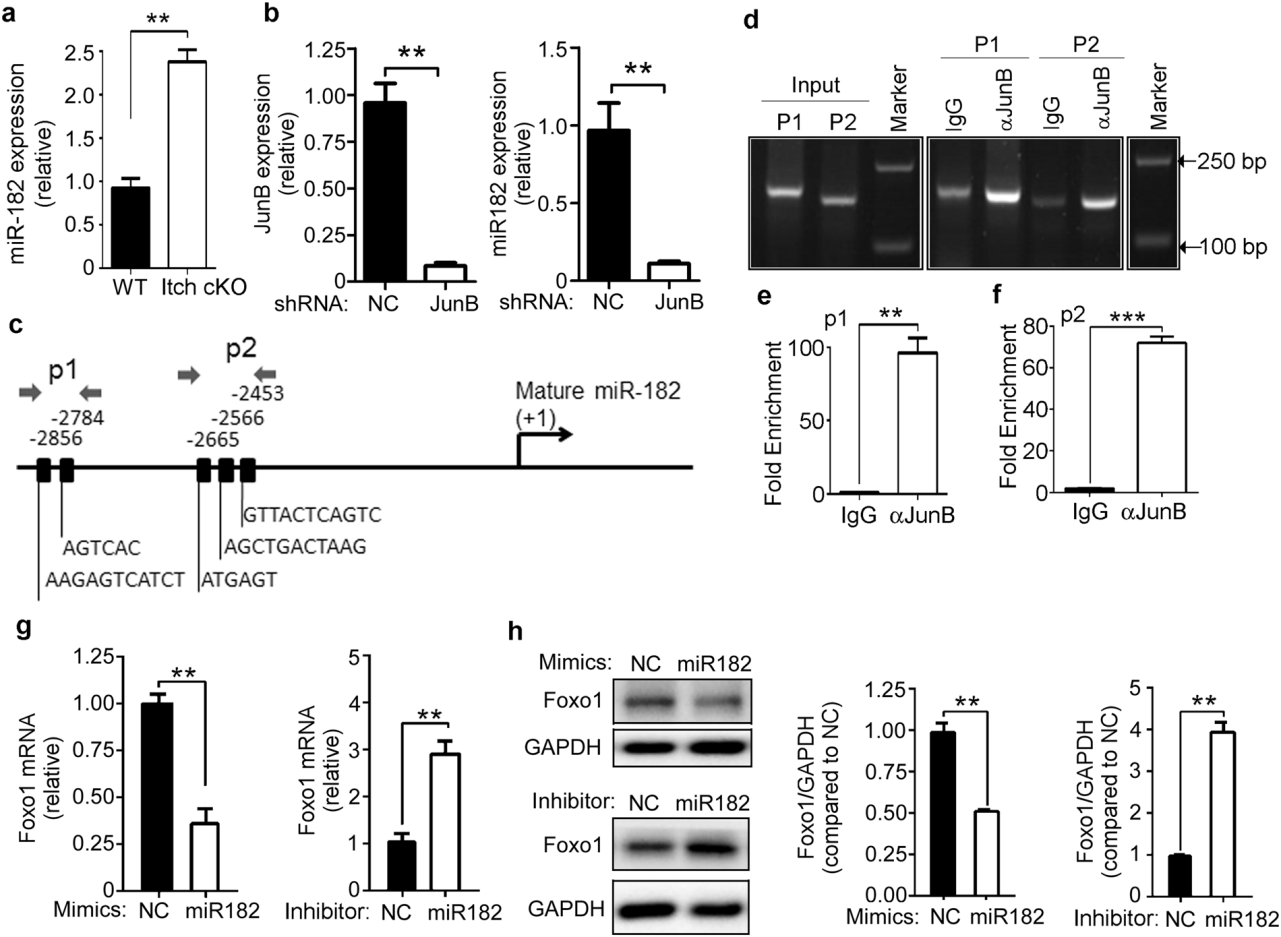
JunB reduces Foxo1 mRNA by up-regulating miR-182. (**a**) Real-time qPCR analysis of miR-182 in B cells from 7~9-week-old Itch cKO mice and WT littermates (n = 3 mice per group, 7~9 weeks old). Relative mRNA levels are normalized to GAPDH mRNA expression and calculated relative to the mRNA expression seen in WT cells, set to 1. (**b**) B cells from 7~9-week-old Itch cKO mice were infected with lentivirus containing negative control (NC) shRNA or JunB-specific shRNA and stimulated with LPS (1 μg/ml). On Day 3, the expression of JunB mRNA and miR-182 was analyzed by qPCR. Relative mRNA levels are normalized to GAPDH mRNA expression and calculated relative to the mRNA expression in NC shRNA-infected cells, set to 1. (**c**) Schematic diagram of mouse miR-182 promoter region illustrating the positions of the primer pairs used for ChIP assays. Sequences represent main predicted JunB binding sites from Supplementary Tables III and IV. Arrows represent the region of the 2 primer pairs. ChIP assays of WT B220^+^ B cells using a JunB antibody or control IgG probing for the miR-182 promoter locus. PCR (**d**) and qPCR using the p1 (**e**) and p2 (**f**) primer pairs were used to analyze the enrichment, and the fold-enrichments are representative of one of four independent experiments. (**g**,**h**) B cells from 7~9-week-old C57BL/6 mice were stimulated with LPS (1 μg/ml) and transduced with miR-182 mimics and inhibitor. On Day 3, the expression of Foxo1 mRNA (**g**) and protein (**h**) was analyzed by qPCR and immunoblotting, respectively. NC: negative control. (**g**) Relative mRNA levels are normalized to GAPDH mRNA expression and calculated relative to the mRNA expression in NC-treated cells, set to 1. (**h**) Band intensities of Foxo1 and GAPDH were quantified using ImageProPlus 5.0 software. The density ratios of Foxo1 to GAPDH compared with the NC group (set as 1) are shown as mean ± SEM (n = 3) of three independent experiments (right panel). (**a–h**) The data represent at least three independent experiments. Two-tailed student’s t-test (**a**,**b**,**e**,**f**) and two-way ANOVA plus Bonferroni post-tests (**g**) to compare each column with control column. Error bars, s.e.m. *p < 0.05, **p < 0.01.

JunB has been reported to up-regulate miR-182, which reduces Foxo1 mRNA expression in zebrafish^25^. Thus, we determined miR-182 expression in B cells from Itch cKO mice and WT littermates by real-time qPCR. Compared with WT mice, Itch cKO mice had a higher level of miR-182 in B cells (Fig. 6a). When JunB was depleted in B cells from Itch cKO using JunB shRNA, miR-182 levels decreased (Fig. 6b). These results suggest that Itch deficiency up-regulates miR-182 by JunB. As a transcription factor, JunB may bind the miR-182 promoter to regulate miR-182 expression. We chose and analyzed the miR-182 promoter sequence (Supplementary Table III). JunB was predicted to have binding sites in the miR-182 promoter (Supplementary Table IV). The main predicted binding sites of JunB were used to design the primer pairs used for ChIP-PCR/qPCR (Fig. 6c). Anti-mouse JunB antibody or control IgG was used to probe the miR-182 promoter locus. The relative binding was defined by qPCR (Fig. 6d–f). The results suggested that JunB can bind the sites amplified by the p1 and p2 primer pairs (Fig. 6d–f).

To prove the role of miR-182 in JunB-reduced Foxo1 mRNA, B cells from C57BL/6 mice were transduced with miR-182 mimics and inhibitor. Compared with the negative control, miR-182 mimics significantly reduced Foxo1 mRNA and protein expression, whereas miR-182 inhibitors significantly up-regulated Foxo1 mRNA and protein expression (Fig. 6g,h). In addition, the alignment of miR-182 with the Foxo1 mRNA 3′-UTR is shown, based on the computed sequence alignment by the TargetScan 5.0 program (Supplementary Table. V). Altogether, these results suggest that JunB reduces Foxo1 mRNA by up-regulating miR-182.

### Foxo1 negatively regulates JunB expression by promoting activation of the Itch promoter

**Figure 7:**
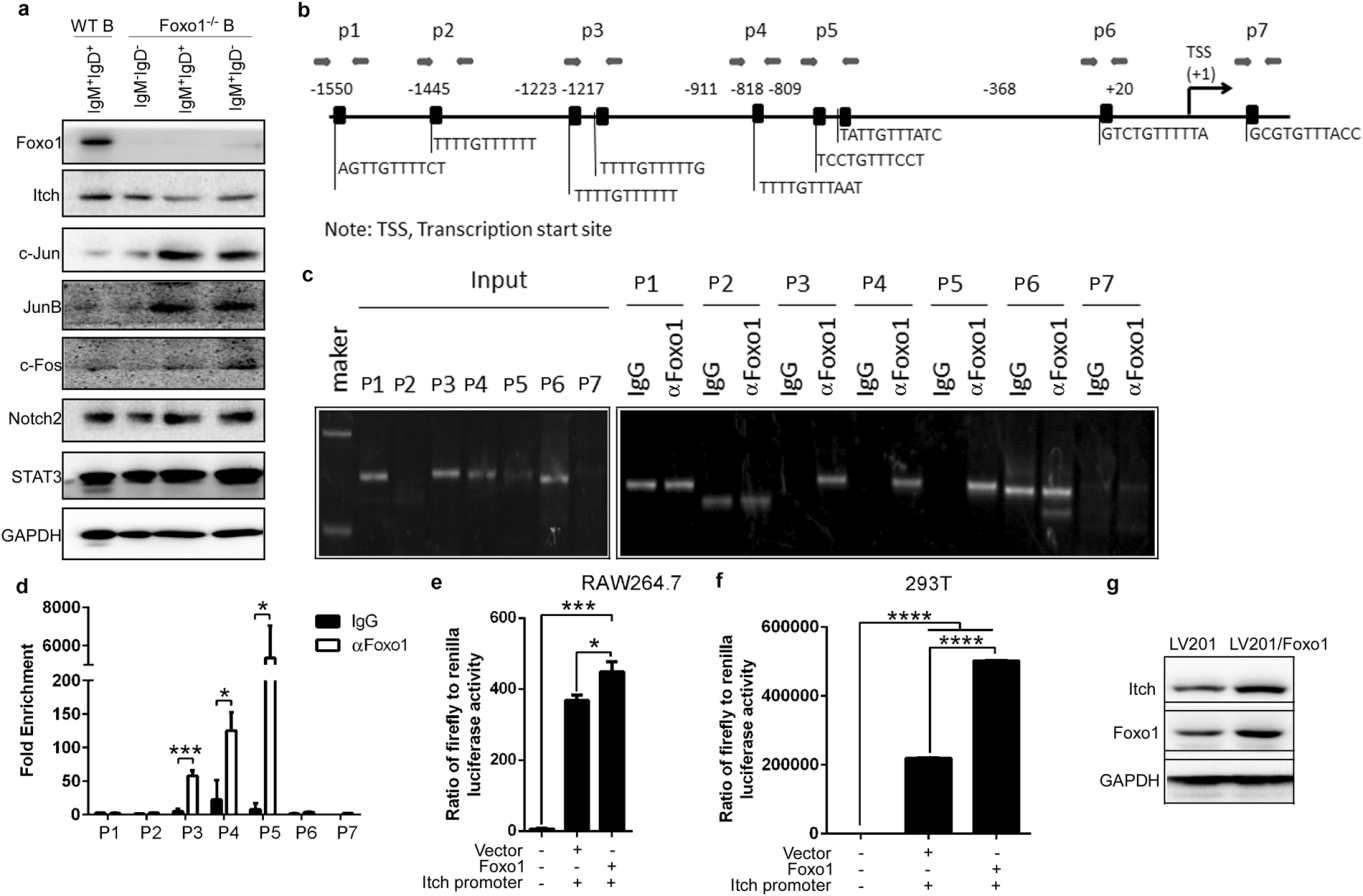
Foxo1 negatively regulates JunB expression by promoting activation of the Itch promoter. (**a**) Immunoblot analysis of Foxo1, Itch, c-Jun, JunB, c-Fos, Notch2, and STAT3 in splenic IgM^+^IgD^+^B cells sorted from 7~9-week-old Itch cKO mice and WT littermates (n = 3 mice per group, 7~9 weeks old). (**b**) Schematic diagram of mouse Itch promoter region illustrating the positions of the primer pairs used for ChIP assays. Sequences represent the main predicted Foxo1 binding sites from Supplementary Tables V and VI. Arrows represent the region of the 7 primer pairs. (**c**,**d**) ChIP assays of WT B220^+^ B cells using a Foxo1 (αFoxo1) antibody or control IgG probing for the Foxo1 promoter locus. PCR (**c**) and qPCR (**d**) were used to analyze the enrichment and the fold-enrichments are representative of one of four independent experiments. (**e**,**f**) Empty vector Lv 201 (Vector) or Lv201/Foxo1 (Foxo1) and luciferase reporter vector pEZX-PG04.1/Itch promoter were co-transduced into RAW264.7 cells (**e**) or 293 T cells (**f**). Dual luciferase reporter gene expression was analyzed, and the results are shown as the ratio of firefly to Renilla luciferase activity. (**g**) Immunoblot analysis of Itch and Foxo1 in lv201 or Foxo1-expressing lv201 lentivirus-infected B cells from 7~9-week-old WT mice. (**a–g**) The data represent at least three independent experiments. Two-tailed student’s t-test (**e**,**f**) and two-way ANOVA plus Bonferroni post-tests (**d**) to compare each column with control column. Error bars, s.e.m. *p < 0.05, ***p < 0.001, ****p < 0.0001.

To explore whether Foxo1 also affects JunB expression, we determined Itch and JunB expression in Foxo1 cKO mice. We found that Foxo1 deficiency reduced Itch, and up-regulated c-Jun, JunB, and c-Fos, whereas Notch2 and STAT3 did not change in splenic IgM^+^IgD^+^B cells (Fig. 7a). These results prompted us to propose that Foxo1 suppresses Itch substrates such as Jun, JunB, and c-Fos by up-regulating Itch expression to promote the ubiquitination and degradation of its substrates. As a transcription factor, Foxo1 may bind the Itch promoter to regulate Itch mRNA expression. We chose and analyzed the Itch promoter sequence (Supplementary Table VI). By using web-based software, we found that Foxo1 has binding sites in the Itch promoter (Supplementary Table VII). The main binding sites were used to design the primer pairs used for ChIP-PCR/qPCR (Fig. 7b). The results suggest that Foxo1 can bind the sites amplified by the p3, p4 and p5 primer pairs (Fig. 7c,d). In addition, our luciferase reporter system demonstrated that Foxo1 significantly effects Itch promoter activation (Fig. 7e,f). Importantly, Foxo1 overexpression in B cells up-regulated Itch expression (Fig. 7g). Altogether, these data suggest that Foxo1 negatively regulates JunB expression by promoting activation of the Itch promoter.

## Discussion

B-cell selection is normally controlled by a series of checkpoints, both centrally in the marrow and in the peripheral lymphoid tissues. B lymphopoiesis requires the concerted action of transcription factors and chromatin-modifying enzymes^26^. Foxo1-dependent genes, such as interleukin 7 receptor α (IL-7Rα), Rag1/Rag2, and CD62L, are critical in the development of pro-B cells, pre-B cells and peripheral B cells, repectively^9^. Thus, as a transcription factor, Foxo1 regulates a serial of events essential for B-cell development. Our data suggest that the E3 ubiquitin ligase Itch has a nonredundant function in many of these Foxo1-regulated events. Thus, we have identified a previously unknown and critical function for Itch in B-cell development.

Itch deficiency promotes Th2 differentiation by reducing the ubiquitination and degradation of JunB^21^. This might contribute to the chronic inflammatory diseases and constant itching in Itch^−/−^ mice^27^. However, Itch^−/−^ mice unexpectedly have a substantial defect in Tfh cell differentiation in response to viral infection^18^. In line with the reduced effects of B cells mediated by Itch^−/−^Tfh cells^18^, we found that Itch cKO mice had a substantial defect in B-cell development. A previous study has shown that higher B-cell activating factor levels at birth are negatively related to allergy development^28^. Thus, having fewer mature B cells may be beneficial in certain chronic inflammatory responses.

Based on the differential expression of cell surface markers, B220^+^CD43^+^ pro-B cells can be further subdivided into 3 stages of development: pre-pro-B, early-pro-B, and late-pro-B cells^9^. IL-7 and its receptor have a central role in controlling the survival, proliferation and differentiation of pro-B cells. The importance of IL-7 in early B-cell development has been further documented by *in vitro* and *in vivo* neutralization studies with anti-IL-7 mAbs^29,30^, and more recently in IL-7Rα and IL-7 knockout (KO)^3^ (3) mice^31,32^. The absence of the IL-7 signal in mice results in the arrest of B-cell development at the pro-B-cell stage^33^. Due to low IL-7Rα levels, Foxo1^L/L^mb1^Cre^ mice have significantly lower percentages of pro-B cells that were CD19^+^BP1^−^ (early-pro-B) and CD19^+^BP1^+^ (late-pre-B) but a higher percentage of CD19^−^BP1^−^ (pre-pro-B) cells^9^. Our data demonstrated that CD19^cre^Itch^F/F^ mice have significantly lower percentages of pro-B (B220^+^CD43^+^CD19^+^) cells, including early-pro-B and late-pre-B B cells, in BM by down-regulating Foxo1-mediated IL-7Rα expression. Thus, Itch plays an important role in Foxo1-dependent IL-7Rα-mediated pro-B development.

In developing B cells, pre-B cell receptor (pre-BCR) signals initiate immunoglobulin light (Igl) chain gene assembly, leading to RAG-mediated DNA double-strand breaks (DSBs)^34^. Intriguingly, due to decreased Rag expression and heavy chain gene rearrangement at the pro-B cell stage, a prominent small resting pre-B (IgM^−^IgD^−^) cell population transits to the periphery and is present in the peripheral blood and spleen in Foxo1^L/L^CD19^Cre^ mice^9^. Our data demonstratethat CD19^cre^Itch^F/F^ mice have significantly higher in the percentages of small resting pre-B (IgM^−^IgD^−^) cells in the spleen, PBMCs and LNs by down-regulating Foxo1-mediated RAG expression. Thus, Itch plays an important role in Foxo1-dependent RAG-mediated pre-B development.

The adhesion molecule L-selectin (CD62L) is a leukocyte homing receptor that has a prominent role in controlling the recirculation and distribution of leukocyte subsets within non-inflamed and inflamed tissues^12,35^. L-selectin supports the dynamic rolling and tethering of B cells and naïve and central memory T cells along the high endothelial venules of peripheral lymph nodes (PLNs)^36^. Due to decreased CD62L expression, Foxo1^L/L^CD19^Cre^ mice have low levels of B cells in LNs^9^. Our data demonstrate that CD19^cre^Itch^F/F^ mice have significantly more B cells with low CD62L expression in PBMCs and fewer B cells in LNs by down-regulating Foxo1-mediated CD62L expression. Thus, Itch plays an important role in Foxo1-dependent CD62L-mediated B migration.

Itch plays a critical role in multiple stages of B-cell differentiation by mediating Foxo1 expression. Itch is associated with the transcription factor Foxo1 and promotes its ubiquitination and degradation, and acts as an essential positive regulator in the differentiation of Tfh cells^18^. However, CD19^cre^Itch^F/F^ mice unexpectedly showed a substantial reduction in Foxo1 expression in B cells. The low Foxo1 expression in B cells resulting from Itch deficiency may not be through ubiquitination but an unknown mechanism. The identification of c-Jun and JunB as two Itch protein substrates^21,37^ has shed light on the molecular basis underlying the immunological phenotype of Itchy mice. As a result of Itch-mediated canonical ubiquitylation of its substrate, JunB, IL-4 promoter occupancy by this transcription factor is greatly reduced upon T-cell receptor (TCR) stimulation^21^. JunB was recently identified as a substrate of Nedd8 modification by Itch^38^. JunB neddylation mediated by Itch attenuates its transcriptional activity and promotes its ubiquitination-dependent degradation^38^. As expected, Itch-deficient B cells had a higher level of JunB.

To determine the activity of JunB on the Foxo1 promoter, we used ChIP-PCR/qPCR technology. Compared with the non-specific binding by the IgG control, the binding site by anti-JunB was significantly amplified by the p1 primer pairs. These results suggest that JunB directly promotes activation of the Foxo1 promoter. Thus, we proposed that Foxo1 mRNA is reduced by JunB on post-transcriptional but not transcriptional level. The JunB-induced microRNA miR-182 attenuates Foxo1 expression, which has been reported to be required for proper lymphatic vascular development in zebrafish^25^. As a consequence of the JunB-mediated dysregulation of miR-135b and miR-194, the oncogene Gα12gep inhibits Foxo1 in hepatocellular carcinoma^24^. Our data show that JunB reduces Foxo1 by promoting miR-182 expression in Itch-deficient B cells. MicroRNAs are small RNA molecules that regulate gene expression and play critical roles in B-cell development and malignancy^39^. As the most highly induced miR^40^, miR-182 plays a critical role in driving extrafollicular B-cell antibody responses. We found that JunB-up-regulated miR-182 suppresses Foxo1 expression, which is required at various stages of B-cell differentiation^9^. A recent study has shown that miR-182-96-183 cluster miR-31, miR-155, miR-150, miR-127, and miR-379 is greatly upregulated in both purified splenic B and T cells in lupus-prone MRL-lpr mice^41^. The miRNA-183-96-182 cluster promotes T helper 17 cell pathogenicity by negatively regulating Foxo1 expression^42^. It is worthwhile to further explore the role of miR-182 in B-cell-related diseases.

A genome-wide analysis of Foxo1 binding sites has revealed ~300 Foxo1-bound target genes, including Itch^43^. We further proved that Foxo1 binds the Itch promoter and promotes its transcription. Thus, Foxo1 suppresses the effect of JunB-mediated miR-182 on Foxo1 expression by enhancing Itch-mediated JunB ubiquitination and degradation in B cells.

Due to Itch deficiency, JunB is up-regulated to promote the expression of miR-182 which bind 3′-UTR of Foxo1 and reduce Foxo1 mRNA. Thus, Itch deficiency affects multiple stages of B-cell differentiation by down-regulating Foxo1 downstream molecules (RAG, IL-7Ra, CD62L, Itch, etc.). On the other hand, Foxo1 induces JunB ubiquitination to reduce the effect of JunB-mediated miR-182 on Foxo1 by up-regulating Itch. In conclusion, we have found a novel regulatory axis between Itch-controlled JunB and Foxo1 expression in B cells (Supplementary Figure 7), which explains why Itch is required for B-cell development.

## Methods and Materials

### Mice

Seven-to-nine-week-old C57BL/6J (B6) mice were purchased from Huafukang Corp., Beijing, China. Mice expressing Cre recombinase under control of the CD19 promoter (CD19^cre^) and mice with loxP sites flanking exon 2 of Foxo1 (Foxo1^F/F^) on the B6 background have been described^44^. Foxo1^F/F^ mice were crossed to CD19^cre^ mice to delete Foxo1 in B cells. The floxed Itch (Itch^F/F^) mice on a B6 background were generated by Shanghai Biomodel Organism Science & Technology Development Co., Ltd (Shanghai, China). Itch^F/F^ mice were crossed to CD19^cre^ mice to delete Itch in B cells. The breeding scheme of the Itch and Foxo1 double conditional knockout mice is described in Supplementary Figure 2c. Care, use, and treatment of mice in this study were in strict agreement with international guidelines for the care and use of laboratory animals. This study was approved by the Animal Ethics Committee of the Beijing Institute of Basic Medical Sciences.

### Immunoprecipitation and immunoblot analysis

Proteins were immunoprecipitated by incubation of the cell lysates overnight at 4 °C with the appropriate antibodies (1 μg, listed in Supplementary Table VIII), followed by the addition of protein A/G–Magnetic beads (88802; Thermo Scientific) and incubation for another 2 h at 4 °C. Immunoprecipitates were washed five times with NP-40 lysis buffer and were boiled in 50 μl SDS loading buffer. Precipitates were washed five times with NP-40 lysis buffer and were boiled in 50 μl SDS loading buffer. For visualization of ubiquitinated protein, 1.0% SDS was added to lysis buffer for disruption of nonspecific protein interactions. Cell lysates were denatured by being boiled for 15 min and then were diluted to a concentration of 0.1% SDS before immunoprecipitation. Samples were separated to 10–12% SDS-PAGE, followed by electrotransfer to PVDF membranes (Millipore). Membranes were analyzed by immunoblot with the appropriate antibodies (listed in Supplementary Table VIII), followed by horseradish peroxidase-conjugated second antibody IgG (H + L) (GE Healthcare, San Francisco, CA) were used in concert with the ECL detection system (Amersham, Arlington Heights, IL).

### Flow cytometry analysis

Cytometric analysis has been described in our previous studies^45,46^. Briefly, cells (1 × 10^6^ cells/sample) were washed with fluorescence-activated cell sorting staining buffer (phosphate-buffered saline, 2% fetal bovine serum or 1% bovine serum albumin, 0.1% sodium azide). All samples were incubated with anti-Fc receptor Ab (BD Biosciences), prior to incubation with other Abs diluted in fluorescence-activated cell sorting buffer supplemented with 2% anti-Fc receptor Ab. Cells were then stained with antibodies listed in Supplementary Table VIII. The samples were filtered immediately before analysis or cell sorting to remove any clumps. Data collection and analyses were performed on a FACS Calibur flow cytometer using CellQuest software.

### Cell sorting

B220^+^ B cells were separated by B220 microbeads (Miltenyi Biotec, Germany) from the spleen and mesenteric and inguinal lymph nodes (LN) of 7–9-week-old female or male C57BL/6, or Itch cKO mice and their WT littermates. Multicolor flow cytometry was performed by gating on CD3^−^CD4^−^B220^+^CD19^+^ cells that were IgM^+^IgD^+^, IgM^+^IgD^−^, IgM^−^IgD^+^ or IgM^−^IgD^−^. All flow cytometry data were acquired with FACSCanto, FACSCantoII, or FACSAria (BD Biosciences), gated on live lymphocyte-sized cells on the basis of forward and side scatter, and analyzed using FlowJo software (Tree Star, Ashland, OR).

### Quantitative PCR analysis

Quantitative PCR analysis has been described in our previous studies^47,48^. Briefly, total RNA was extracted from B cells with Trizol (Invitrogen Life Technologies). The final RNA pellets were dissolved in 0.1 mM EDTA (2 μl/mg original wet weight). Reverse transcription reactions were carried out on 22 μl of sample using superscript II RNAse H-Reverse Transcriptase (Invitrogen Life Technologies) in a reaction volume of 40 μl. All samples were diluted in 160 μl nuclease-free water. qPCR was employed to quantify mouse gene expression from the cDNA samples. Mouse gene expression was normalized to the levels of the β-actin gene. Sequences of primer pairs are listed in Supplementary Table IX.

### JunB-specific shRNA infected B cells

B cells were infected with shRNA using standard methods as described in our previous studies^44,47,48^. Briefly, in a six well tissue culture plate, seed 1 × 10^6^ B cells per ml in 2 ml antibiotic-free normal growth medium supplemented with FBS. Cells were stimulated overnight with LPS (1 μg/ml, L2880, Sigma-Aldrich, St. Louis, MO, USA) at 37 °C in a CO_2_ incubator. On day 2, 1 × 10^6^ infectious units of virus (IFU) of negative control (N.C), JunB-specific shRNA-expressing lentivirus (sc-35727-V, Santa Cruz Biotech, USA) and 10 μg/ml polybrene (H9268, Sigma-Aldrich, St.) were added into the culture. On day 1 after infection, the transfection mixture was removed and 1x normal growth medium containing LPS (1 μg/ml) was added into the culture.

### Prediction of transcription factor binding sites of Foxo1, miR-182 and Itch promoter

We used the web (http://www.cbs.dtu.dk/services/Promoter/) to identify potential promoter sequences of Foxo1, miR-182 and Itch gene listed in Supplementary Tables I, III, V, repectively. As expected, the sequences have obvious characteristics of promoter. Subsequently, we used the web http://jaspar.genereg.net/ to predict the binding sites of JunB on the Foxo1 promoter, JunB on the miR-182 promoter, and Foxo1 on the Itch promoter listed in Supplementary Table II, IV, VI, respectively.

### Chromatin Immunoprecipitation (ChIP)-PCR/qPCR

Chromatin was immunoprecipitated according to the manufacturer’s instruction (#9002, Cell Signaling). Briefly, sorted cells were crosslinked with 1% (vol/vol) formaldehyde at room temperature for 10 min, and incubated with glycine for 5 min at room temperature. Cells were then sequentially washed in ice-cold buffer A and buffer B, followed by digesting with MNase. Nuclear pellet was suspended in ChIP buffer, sheared by sonication with an average size of sheared fragments of about 300 base pairs (bp) to 800 bp. After centrifugation at 10,000 rpm for 10 minutes, sheared chromatin was diluted in ChIP buffer and precleared by addition of protein A/G plus agarose beads (sc-2003) for 1 h at 4 °C. Before antibody incubation, input samples were removed from the lysate and stored at −80 °C until extraction. The beads were discarded and the supernatant was then incubated with anti-mouse Foxd3 antibody (sc-133588, Santa Cruz Biotech) or control anti-IgG (Cell Signaling Tech), at 4 °C overnight. At the next day, protein A/G plus agarose beads were added and incubated for 2 h at 4 °C. Beads were harvested by centrifuge and went through 3 low salt washes and one high salt wash. Beads were then eluted with ChIP elution buffer. The elutes and input were then added with proteinase K and RNase A and heated at 65 °C for 2 h to reverse the formaldehyde cross-link. DNA fragments were purified with Chip DNA clean & concentrator^TM^-capped column (D5205, ZYMO Research Corp, CA, USA). The immunoprecipitated and input DNA, and A SYBR Green PCR kit (Bio-Rad) were used for quantitative real-time PCR analysis. PCR was conducted on an initial denaturing step of 3 minutes at 94 °C followed by 45 cycles of 94 °C for 10 seconds, 60 °C for 15 seconds and 72 °C for 10 seconds and then a final extension at 72 °C for seven minutes. The results were quantified with an Icycler IQ (Bio-Rad). The relative binding was defined by determining the immunoprecipitation level (ratio of the amount of immunoprecipitated DNA to that of the input sample).

### Foxo1 and Itch promoter reporting gene analysis

The firefly luciferase reporter plasmid pEZX-PG04.1 with the 5′-flanking region from start codon upstream −1850∼−154 of mouse Foxo1 gene (MPRM18683-PG04) and −1567∼+9 of mouse Itch gene (MPRM36377-PG04), Empty vector Lv201 and plasmids Lv201 expressing Foxo1 (EX-Mm07646-Lv201) or JunB (EX-Mm03394-Lv201) were purchased from were purchased from GeneCopoeia^TM^, Rockville, MD, USA. To ensure that the recombinant clone, we isolated the vector and verified that no mutations were introduced during cloning by DNA sequencing and that the construct was in the correct orientation. 0.5 μg Lv201/Foxo1 or Lv201/JunB, 0.5 μg firefly luciferase reporter plasmids pEZX-PG04.1/Foxo1 promoter or pEZX-PG04.1/Itch promoter, and 0.05 μg Renilla luciferase reporter vector pRL-SV-40 vector (cat# E2231, Promega Corp.) were co-transduced into 4 × 10^5^ RAW264.7 or 293T cells in 12-well plate by using 6 μL Lipofectamine®2000 Reagent (Cat# 11668-019, Invitrogen Corp.). On day 3, sequential measurement of firefly luciferase (Reporter #1) followed by Renilla luciferase activity (Reporter #2) was assessed on 1420 Multilabel Counter (1420 Victor 3, PerkinElmer Corp.), and analyzed. The results were shown as the ratio of firefly to Renilla luciferase activity.

### Real-time PCR assays for miRNAs

For quantitative PCR of mature miRNA, we routinely isolate total RNA from cultured or sorted cells using TRIZOL (Invitrogen, Carlsbad, CA) per the manufacturer’s protocol. Total RNA is briefly exposed to RNAase-free DNAase I. RNA is reverse transcribed to cDNA using a gene specific primer (listed in Supplementary Table IX) and Thermoscript, thermostable reverse transcriptase (Invitrogen). A 10.5 µl reaction was assembled using 10 µM of the anti-sense primer and a primer for the internal control (typically U6). The reaction was heated to 80 °C for 5 min to denature the RNA, followed by a 5 min incubation at 60 °C to anneal the primers. The reactions were cooled to room temperature and the remaining reagents (5 × buffer, dNTPs, DTT, RNase inhibitor, Thermoscript) were added as specified in the Thermoscript protocol. The reaction proceeded for 45 min at 60 °C followed by a 5 min incubation at 85 °C to inactivate the Thermoscript RT. cDNA may be stored indefinitely at −20° or −80 °C. The generated cDNA was amplified using PCR in the reaction mixtures containing 2 × QuantiTect SYBR Green PCR master mix and miRNA-specific primers (listed in Supplementary Table IX). For normalization, transcripts of U6 small RNA were used as control.

### Transfection with miRNAs or antisense oligonucleotides

Synthetic miRNA duplexes were used and listed in Supplementary Table IX. Cells were transiently transfected with 20 nM or 60 nM of control mimic (Dharmacon) or miR-182 mimic; or 20 nM or 100 nM of miR-182 inhibitor or respective negative control RNA using FuGENE® HD Reagent.

### Foxo1-expressing lentivirus infected B cells

B cells were infected with lentivirus using standard methods as described in our previous studies^46,49^. Briefly, in a six well tissue culture plate, seed 1 × 10^6^ B cells per ml in 2 ml antibiotic-free normal growth medium supplemented with FBS. Cells were stimulated overnight with LPS (20 μg/ml, L2880, Sigma-Aldrich, St. Louis, MO, USA) at 37 °C in a CO_2_ incubator. On day 2, 1 × 10^6^ infectious units of virus (IFU) of EGFP-expressing lentivirus (negative control, LV201), Foxo1- and EGFP-expressing lentivirus (LV201/Foxo1, Fugene Corp., Guangzhou, China) and 10 μg/ml polybrene (H9268, Sigma-Aldrich, St.) were added into the culture. On day 1 after infection, the transfection mixture was removed, 1x normal growth medium containing LPS (20 μg/ml) was added into the culture and cells were re-stimulated for 2 days. EGFP^+^ cells were sorted by FACS and analyzed by western blot.

### Statistics

Statistics were analyzed by using GraphPad Prism (version 5.0, GraphPad Software Inc., USA). The data were shown as mean ± standard error of the mean (SEM). Student’s t test was employed to determine significance between two groups (paired or unpaired) and One-Way and Two-Way ANOVA analysis was used to determine significance among several groups. Differences were considered statistically significant when p < 0.05.

## Electronic supplementary material

Supplementary Materials

## References

[1] Pieper K, Grimbacher B, Eibel H (2013). B-cell biology and development. J. Allergy Clin. Immunol..

[2] Abolhassani H, Parvaneh N, Rezaei N, Hammarstrom L, Aghamohammadi A (2014). Genetic defects in B-cell development and their clinical consequences. J. Investig. Allergol. Clin. Immunol..

[3] Conley ME (2002). Early defects in B cell development. Curr. Opin. Allergy Clin. Immunol..

[4] Jiang XX (2011). Control of B cell development by the histone H2A deubiquitinase MYSM1. Immunity.

[5] Clark MR, Mandal M, Ochiai K, Singh H (2014). Orchestrating B cell lymphopoiesis through interplay of IL-7 receptor and pre-B cell receptor signalling. Nat. Rev. Immunol..

[6] Rolink AG, Schaniel C, Busslinger M, Nutt SL, Melchers F (2000). Fidelity and infidelity in commitment to B-lymphocyte lineage development. Immunol. Rev.

[7] Tudor KS, Payne KJ, Yamashita Y, Kincade PW (2000). Functional assessment of precursors from murine bone marrow suggests a sequence of early B lineage differentiation events. Immunity.

[8] Milne CD, Paige CJ (2006). IL-7: a key regulator of B lymphopoiesis. Semin. Immunol..

[9] Dengler HS (2008). Distinct functions for the transcription factor Foxo1 at various stages of B cell differentiation. Nat. Immunol..

[10] Ley K (1991). Lectin-like cell adhesion molecule 1 mediates leukocyte rolling in mesenteric venules *in vivo*. Blood.

[11] Kansas GS, Ley K, Munro JM, Tedder TF (1993). Regulation of leukocyte rolling and adhesion to high endothelial venules through the cytoplasmic domain of L-selectin. J. Exp. Med..

[12] Ley K, Kansas GS (2004). Selectins in T-cell recruitment to non-lymphoid tissues and sites of inflammation. Nat. Rev. Immunol..

[13] Gallatin WM, Weissman IL, Butcher EC (1983). A cell-surface molecule involved in organ-specific homing of lymphocytes. Nature.

[14] Steeber DA, Green NE, Sato S, Tedder TF (1996). Lymphocyte migration in L-selectin-deficient mice. J. Immunol..

[15] Loder F (1999). B cell development in the spleen takes place in discrete steps and is determined by the quality of B cell receptor-derived signals. J. Exp. Med..

[16] Szydlowski M, Jablonska E, Juszczynski P (2014). FOXO1 transcription factor: a critical effector of the PI3K-AKT axis in B-cell development. Int. Rev. Immunol..

[17] Mansson R (2012). Positive intergenic feedback circuitry, involving EBF1 and FOXO1, orchestrates B-cell fate. Proc. Natl. Acad. Sci. USA.

[18] Xiao N (2014). The E3 ubiquitin ligase Itch is required for the differentiation of follicular helper T cells. Nat. Immunol..

[19] Giamboi-Miraglia A (2015). The E3 ligase Itch knockout mice show hyperproliferation and wound healing alteration. FEBS J..

[20] Parravicini V, Field AC, Tomlinson PD, Basson MA, Zamoyska R (2008). Itch−/− alphabeta and gammadelta T cells independently contribute to autoimmunity in Itchy mice. Blood.

[21] Fang D (2002). Dysregulation of T lymphocyte function in itchy mice: a role for Itch in TH2 differentiation. Nat. Immunol..

[22] Jin HS, Park Y, Elly C, Liu YC (2013). Itch expression by Treg cells controls Th2 inflammatory responses. J. Clin. Invest..

[23] Zhang M (2007). Ubiquitinylation of Igb dictates the endocytic fate of the B cell antigen receptor. J Immunol..

[24] Jung HS (2014). Ga12gep oncogene inhibits FOXO1 in hepatocellular carcinoma as a consequence of miR-135b and miR-194 dysregulation. Cell. Signal..

[25] Kiesow K (2015). Junb controls lymphatic vascular development in zebrafish via miR-182. Sci. Rep.

[26] Choukrallah MA, Matthias P (2014). The Interplay between Chromatin and Transcription Factor Networks during B Cell Development: Who Pulls the Trigger First?. Front Immunol.

[27] Hustad CM (1995). Molecular genetic characterization of six recessive viable alleles of the mouse agouti locus. Genetics..

[28] Lundell AC (2015). Higher B-cell activating factor levels at birth are positively associated with maternal dairy farm exposure and negatively related to allergy development. J. Allergy Clin. Immunol..

[29] Era T (1994). How B-precursor cells are driven to cycle. Immunol. Rev..

[30] Grabstein KH (1993). Inhibition of murine B and T lymphopoiesis *in vivo* by an anti-interleukin 7 monoclonal antibody. J. Exp. Med..

[31] Peschon JJ (1994). Early lymphocyte expansion is severely impaired in interleukin 7 receptor-deficient mice. J. Exp. Med..

[32] von Freeden-Jeffry U (1995). Lymphopenia in interleukin (IL)-7 gene-deleted mice identifies IL-7 as a nonredundant cytokine. J. Exp. Med..

[33] Wei C, Zeff R, Goldschneider I (2000). Murine pro-B cells require IL-7 and its receptor complex to up-regulate IL-7R alpha, terminal deoxynucleotidyltransferase, and c mu expression. J. Immunol..

[34] Bednarski JJ (2016). RAG-mediated DNA double-strand breaks activate a cell type-specific checkpoint to inhibit pre-B cell receptor signals. J. Exp. Med..

[35] Ivetic A (2013). Signals regulating L-selectin-dependent leucocyte adhesion and transmigration. Int. J. Biochem. Cell Biol..

[36] von Andria UH, Mempel TR (2003). Homing and cellular traffic in lymph nodes. Nat. Rev. Immunol..

[37] Oberst A (2007). The Nedd4-binding partner 1 (N4BP1) protein is an inhibitor of the E3 ligase Itch. Proc. Natl. Acad. Sci. USA.

[38] Li H (2016). Itch promotes the neddylation of JunB and regulates JunB-dependent transcription. Cell Signal..

[39] Fernando TR, Rodriguez-Malave NI, Rao DS (2012). MicroRNAs in B cell development and malignancy. J. Hematol. Oncol..

[40] Pucella JN (2015). miR-182 is largely dispensable for adaptive immunity: lack of correlation between expression and function. J. Immunol..

[41] Dai R (2010). Identification of a common lupus disease-associated microRNA expression pattern in three different murine models of lupus. PLoS One.

[42] Ichiyama K (2016). The MicroRNA-183-96-182 Cluster Promotes T Helper 17 Cell Pathogenicity by Negatively Regulating Transcription Factor Foxo1 Expression. Immunity.

[43] Ouyang W (2012). Novel Foxo1-dependent transcriptional programs control T(reg) cell function. Nature.

[44] Liu X (2016). Metabotropic glutamate receptor 3 is involved in B-cell-related tumor apoptosis. Int. J. Oncol.

[45] Xing C (2015). Critical role for thymic CD19^+^CD5^+^CD1d^hi^IL^−^10^+^ regulatory B cells in immune homeostasis. J. Leukoc. Biol..

[46] Ma N (2014). BAFF Suppresses IL-15 Expression in B Cells. J. Immunol..

[47] Ma N (2015). Ligation of metabotropic glutamate receptor 3 (Grm3) ameliorates lupus-like disease by reducing B cells. Clin. Immunol..

[48] Wang X (2016). A novel IL-23p19/Ebi3 (IL-39) cytokine mediates inflammation in lupus-like mice. Eur. J. Immunol..

[49] Zhang Y (2017). CD19 controls the differentiation from marginal zone precursor (MZP) to MZ B cells by regulating ADAM28-mediated Notch2 cleavage. J Cell Mol Med.

