# Supplementary material for: The E3 ubiquitin ligase Itch is required for B-cell development
Source: Sci Rep. 2019 Jan 23;9:421. doi: 10.1038/s41598-018-36844-9 (PMC6344599; doi:10.1038/s41598-018-36844-9)
Supplement: Supplementary file 1 — Supplementary Materials [file 41598_2018_36844_MOESM1_ESM.pdf]

The E3 ubiquitin ligase Itch is required for B-cell development

Xiaoling Liu, Yu Zhang, Yinxiang Wei, Zhiding Wang, Gaizhi Zhu, Ying Fang, Bing Zhai, Ruonan Xu, Gencheng Han, Guojiang Chen, He Xiao, Chunmei Hou, Beifen Shen, Yan Li, Ning Ma, Renxi Wang

Supplementary Figure 1

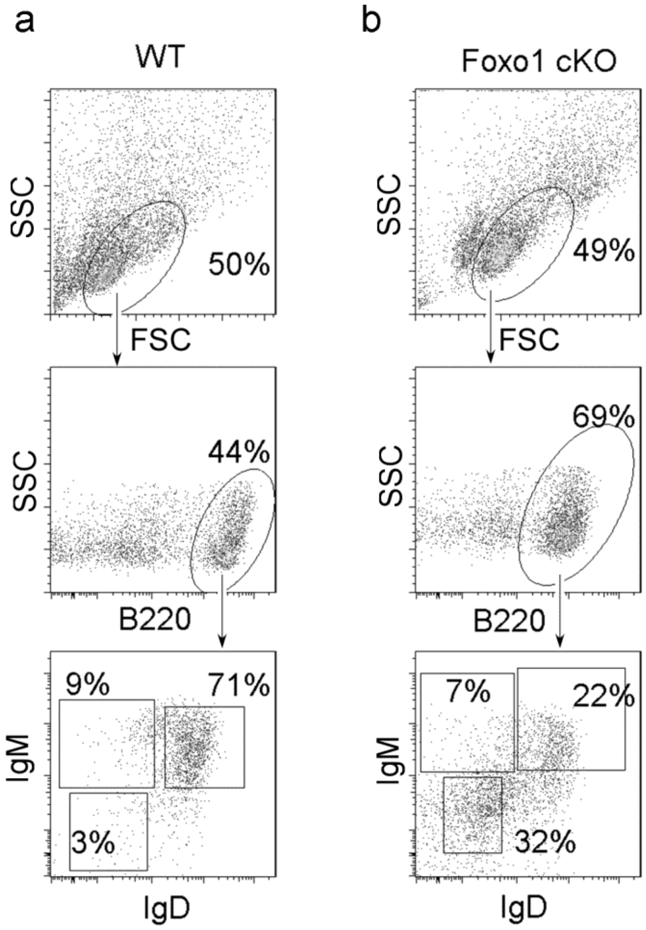

Supplementary Figure 1 The gating strategy for analysis and sorting of splenic B-cell subpopulations. Briefly, splenocytes from 7-9-week-old C57BL/6 mice (Wild type, WT) (**a**) and $CD19^{cre}Foxo1^{F/F}$ (Foxo1 conditional knock out, Foxo1 cKO) (**b**) mice were purified using lymphocytes separate solution. Splenic lymphocytes were staining with anti-mouse B220, IgM, and IgD antibodies. Gated on $B220^{+}$ B cells, mature B cells ($IgM^{+}IgD^{+}$), immature B cells ($IgM^{+}IgD^{-}$), and small resting pre-B cells ($IgM^{-}IgD^{-}$) that transited to the periphery were analyzed and sorted by flow cytometry (FACS). Numbers in quadrants indicate percent cells in each.

Supplementary Figure 2

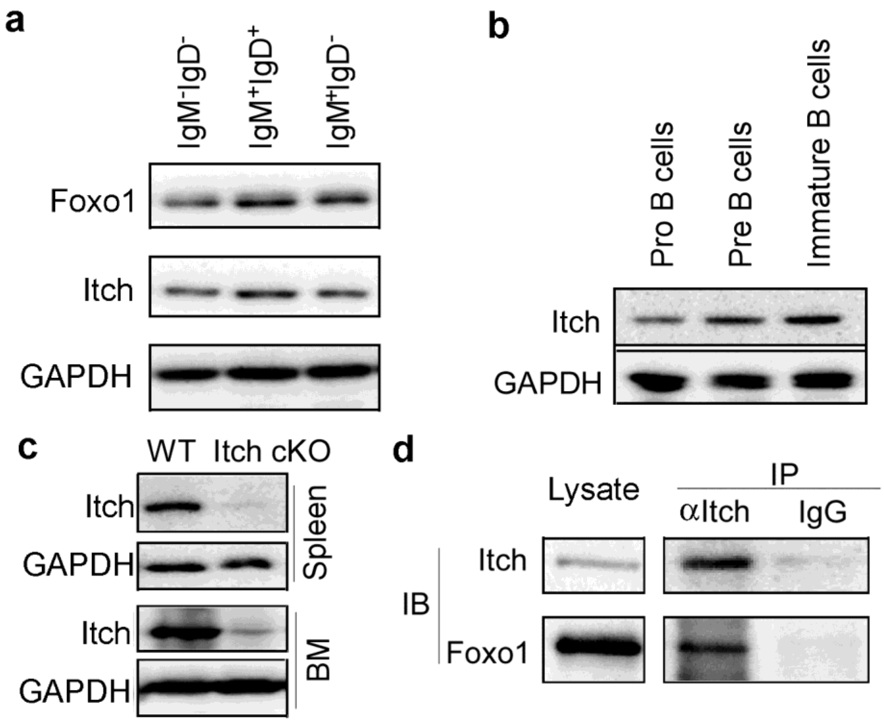

Supplementary Figure 2 Itch protein expression in B cells. Immunoblot analysis in purified mouse splenic B cell populations (IgM^-IgD^-, IgM^+IgD^- and IgM^+IgD^+ B cells) sorted from 7-9-week-old C57BL/6 mice by FACS described in supplementary figure 1a (**a**), purified bone marrow (BM) cell populations ($CD43^+B220^+CD19^+IgM^-$ pro B cells, $B220^+CD19^+CD43^-IgM^-$ pre-B cells and $B220^+CD19^+CD43^-IgM^+IgD^-$ immature B cells) sorted from 7-9-week-old C57BL/6 mice (**b**), and purified B cells sorted from the spleen (upper two panels) and bone marrow (lower two panels) of 7-9-week-old $CD19^{WT}Itch^{F/F}$ (wild type, WT) and $CD19^{Cre}Itch^{F/F}$ (Itch conditional knock out, Itch cKO) mice by using B220 microbeads (**c**). (**d**) Splenic B cells were sorted from 7-9-week-old C57BL/6 mice by using B200 microbeads and subjected to immunoprecipitation with anti-Itch antibodies (αItch) and isotype control IgG (IgG) and immunoblotting with antibodies that were reactive to the indicated proteins.

Aliquots of total cell lysates were similarly immunoblotted. Shown are the results of one representative experiment out of three.

Supplementary Figure 3

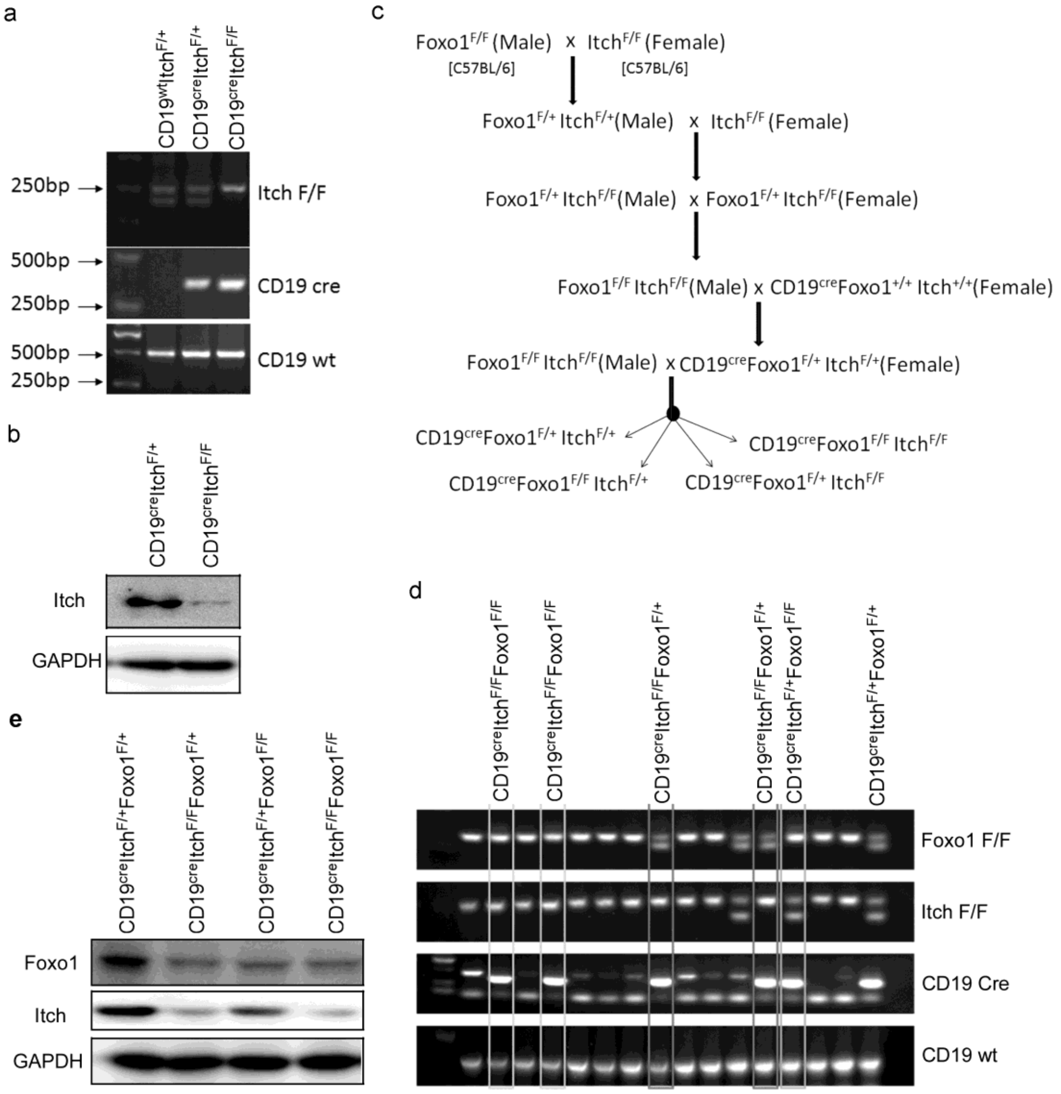

Supplementary Figure 3 Efficiency of deletion of Itch, Foxo1, or Foxo1 and Itch in Cre-systems. (**a**, **d**) Genotype identification of CD19^{WT}Itch$^{F/+}$, CD19^{cre}Itch$^{F/+}$, and CD19^{cre}Itch$^{F/F}$ mice (**a**) and CD19^{cre}Itch$^{F/+}$Foxo1$^{F/+}$, CD19^{cre}Itch$^{F/F}$Foxo1$^{F/+}$, CD19^{cre}Itch$^{F/+}$Foxo1$^{F/F}$, and CD19^{cre}Itch$^{F/F}$Foxo1$^{F/F}$ mice (**d**) by tail-PCR assay. (c) The breading scheme for the double conditional knockout mice. (**b, e**) Western blot analysis for Itch and Foxo1 protein expression from splenic B cells purified from CD19^{cre}Itch$^{F/+}$ and CD19^{cre}Itch$^{F/F}$ mice (**b**), or CD19^{cre}Itch$^{F/+}$Foxo1$^{F/+}$, CD19^{cre}Itch$^{F/F}$Foxo1$^{F/+}$, CD19^{cre}Itch$^{F/+}$Foxo1$^{F/F}$, and CD19^{cre}Itch$^{F/F}$Foxo1$^{F/F}$ mice (**e**).

Supplementary Figure 4

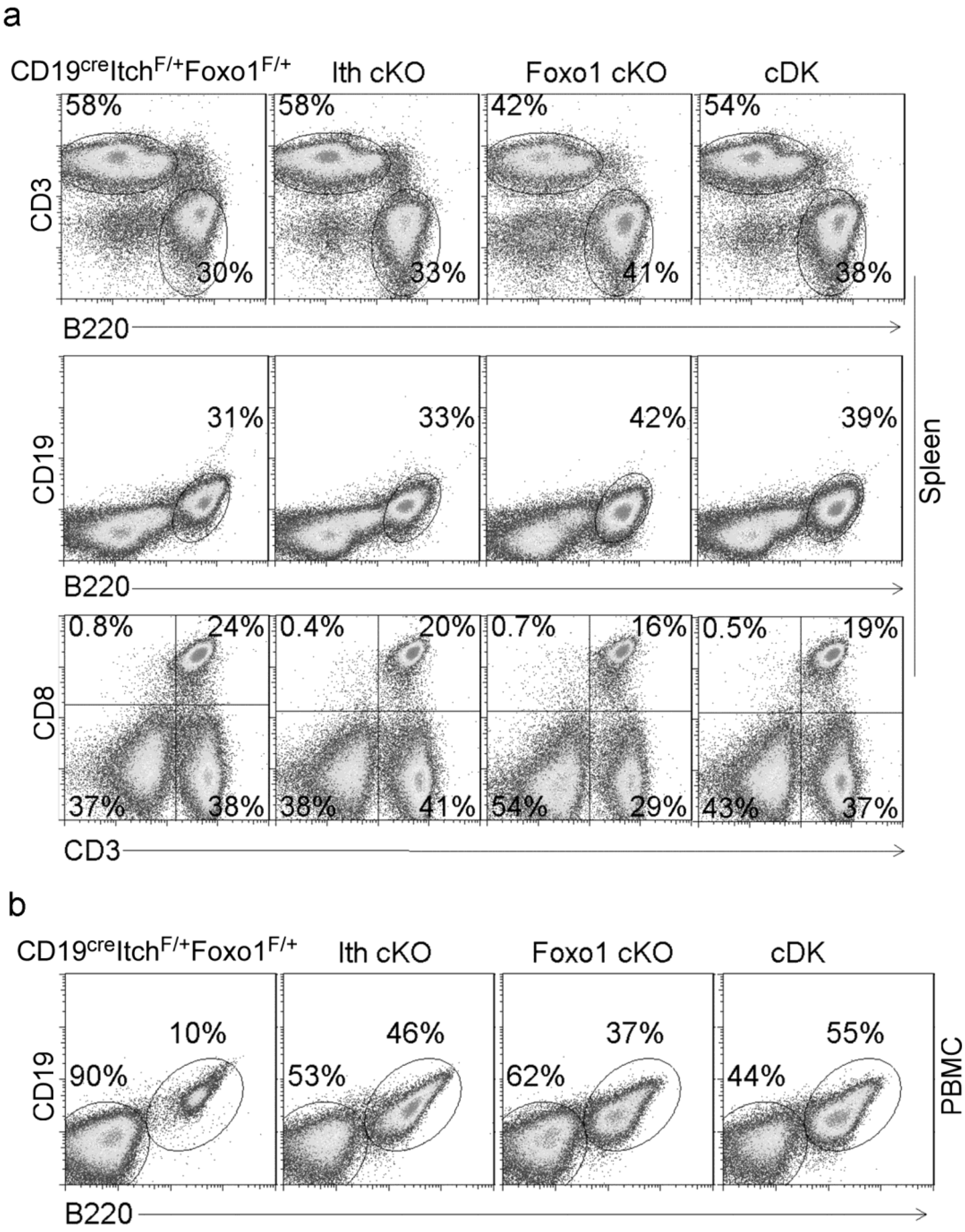

Supplementary Figure 4 B cells increase in PBMC but not spleen from both Itch cKO and Foxo1 cKO. Representative flow cytometry profiles of $CD3^+$T cells, $B220^+$ B cells, $CD19^+B220^+$B cells, and $CD8^+CD3^+$ T cells in spleen (**a**), and $CD19^+B220^+$B cells in PBMC (**b**) from $CD19^{cre}Itch^{F/+}Foxo1^{F/+}$, $CD19^{cre}Itch^{F/F}Foxo1^{F/+}$, $CD19^{cre}Itch^{F/+}Foxo1^{F/F}$, and $CD19^{cre}Itch^{F/F}Foxo1^{F/F}$ mice (n = 6 mice per group, 7~9

weeks old). Numbers in quadrants indicate percent cells in each. Results represent at least five independent experiments (n = 6 per group).

Supplementary Figure 5

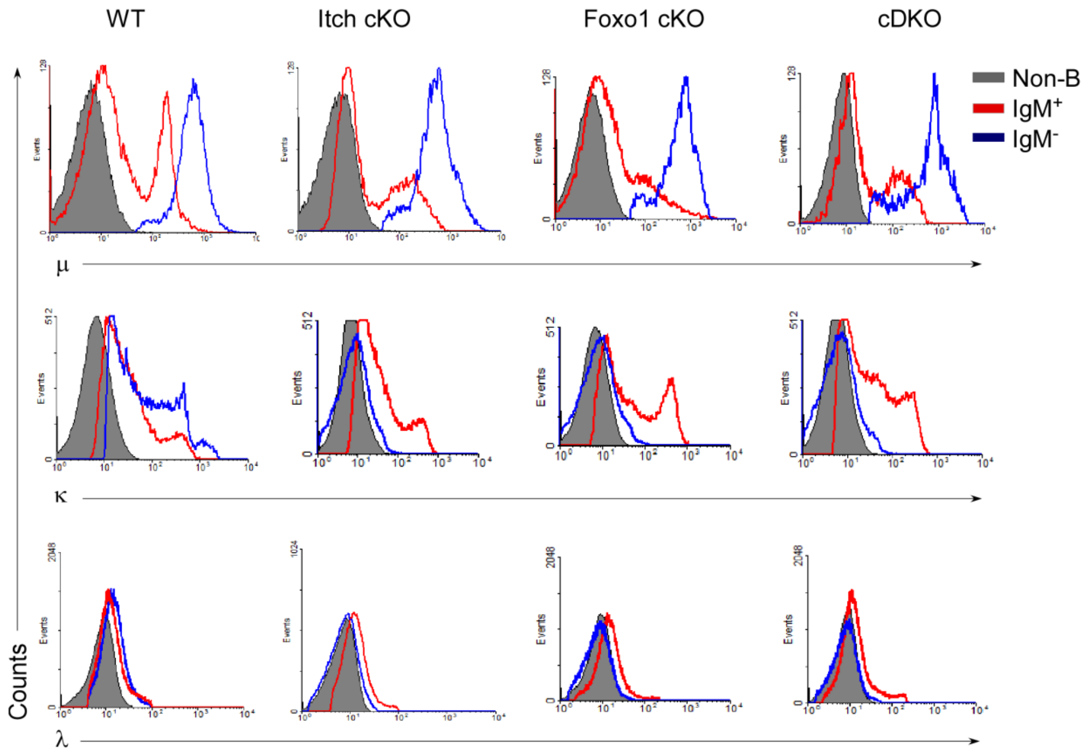

Supplementary Figure 5 $IgM^{-}IgD^{-}$ B cells from Itch cKO, Foxo1 cKO, and Itch-Foxo1 cDK mice are small resting pre-B cells. Expression of intracellular μ heavy chain and κ or λ light chain in splenic IgM^{+} (red), IgM^{-} (blue) B cells, and non-B cells (shaded gray). FACS plots are representative of 3 mice/group.

Supplementary Figure 6

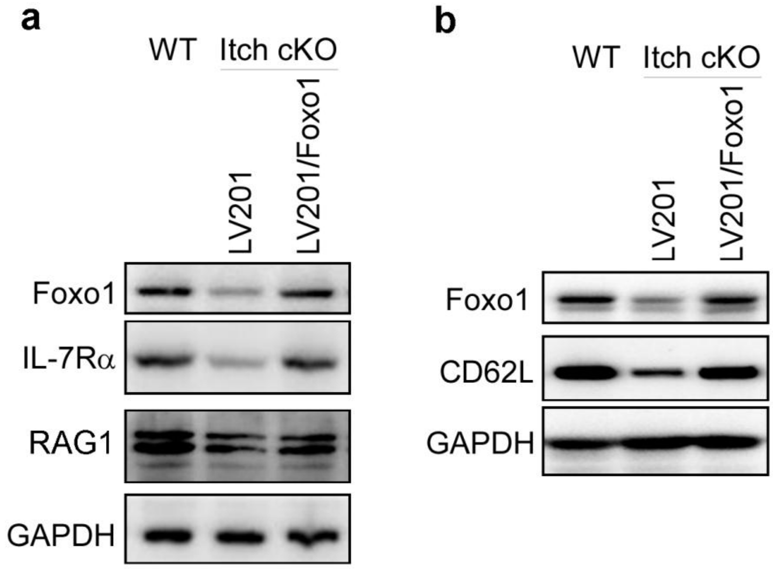

Supplementary Figure 6 Itch regulates RAG, IL-7Rα and CD62L expression via Foxo1. (**a**) BM $CD43^+B220^+CD19^+c\text{-}kit^+$ pro–B cells from Itch cKO and its littermates were sorted by FACS. The whole bone marrow cells were collected and cultured in a culture flask. After 4 days of incubation in a 37C, 5% CO_2 humidified incubator, the nonadherent cells were removed from the cultures by gentle aspiration and the adherent cells were collected as primary stroma cells. 1 x 10^6 pro-B cells per ml and 1 x 10^5 irradiated primary stroma cells per ml were cultured in a six well tissue culture plate in 2 ml antibiotic-free normal growth medium supplemented with FBS in the presence of recombinant mouse IL-7 (100 U/ml). At the same time, 1 x 10^6 infectious units of virus (IFU) of negative control lentivirus (LV201), Foxo1-expressing lentivirus (LV201/Foxo1) and 10 μg/ml polybrene were added into the culture. On day 3 after infection, the cells were collected and analyzed by western blot. Immunoblot analysis of Foxo1, IL-7Rα, and RAG1 was shown (n = 3 mice per group, 7~9 weeks old). (**b**) Splenic B cells from Itch cKO and its littermates were

sorted by CD19 microbeads. 1 x 10^6 B cells per ml were cultured in a six well tissue culture plate in 2 ml antibiotic-free normal growth medium supplemented with FBS. At the same time, 1 x 10^6 infectious units of virus (IFU) of negative control lentivirus (LV201), Foxo1-expressing lentivirus (LV201/Foxo1) and 10 μg/ml polybrene were added into the culture. On day 3 after infection, cells were collected and analyzed by western blot. Immunoblot analysis of Foxo1 and CD62L was shown (n = 3 mice per group, 7~9 weeks old).

Supplementary Figure 7

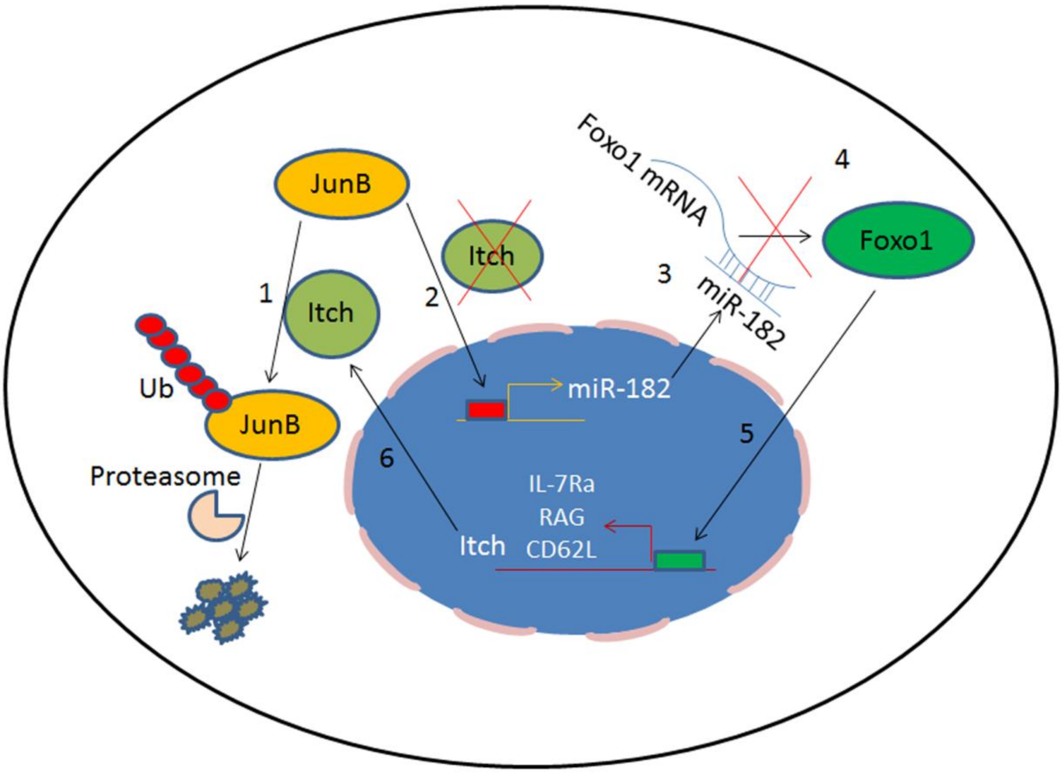

Supplementary Figure 7 Scheme for novel regulatory axis in Itch-controlled Foxo1 expression in B cells. 1, Itch is molecularly associated with and induced ubiquitination of JunB which serves as a signal for proteasome- or lysosome-mediated degradation; 2, When Itch is deficient, JunB protein is up-regulated, enters into the nuclear, binds miR-182 promoter and as thus, miR-182 is up-regulated; 3, miR-182 destroyed Foxo1 mRNA by binding 3’-UTR of Foxo1. 4, Low copies of mRNA result in low level of Foxo1 protein; 5, Foxo1 enters into the nuclear and binds the promoters of its downstream molecules (RAG, IL-7Ra, CD62L, Itch, etc.); 6, Foxo1 induces ubiquitination of JunB by up-regulating Itch.

Supplementary Figure 8

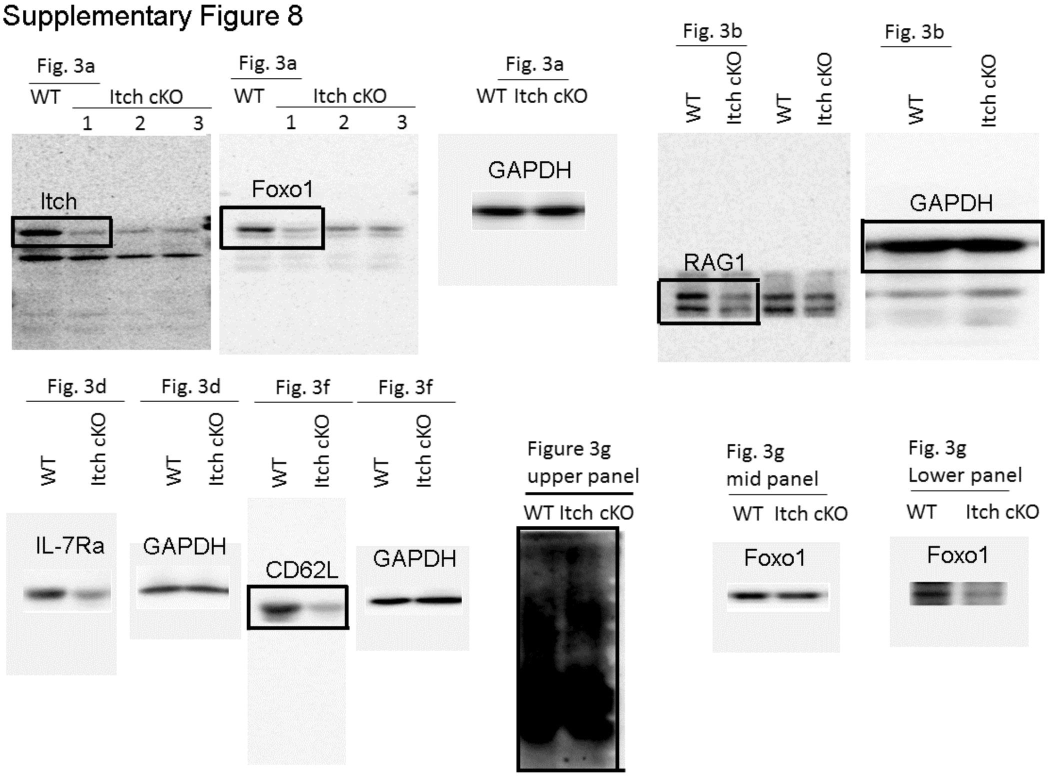

Supplementary Figure 8 original scans for Figure 3a, 3b, 3d, 3f, and 3g.

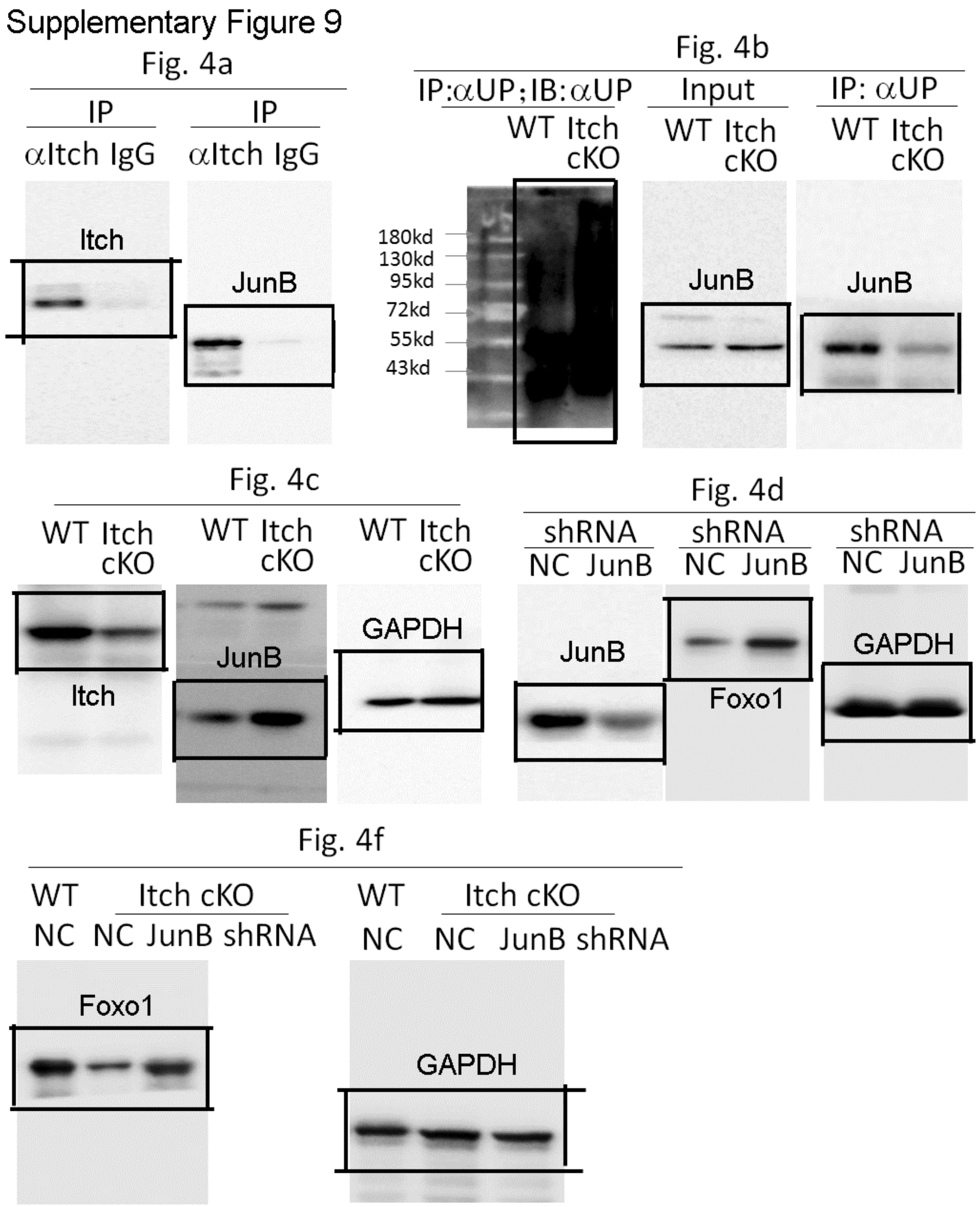

Supplementary Figure 9 original scans for Figure 4a, 4b, 4c, 4d, and 4f.

Supplementary Figure 10

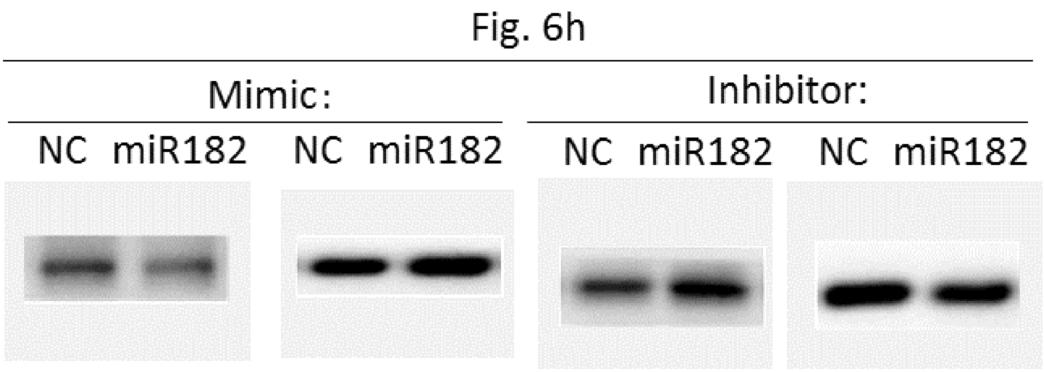

Supplementary Figure 10 original scans for Figure 6h.

Supplementary Figure 11

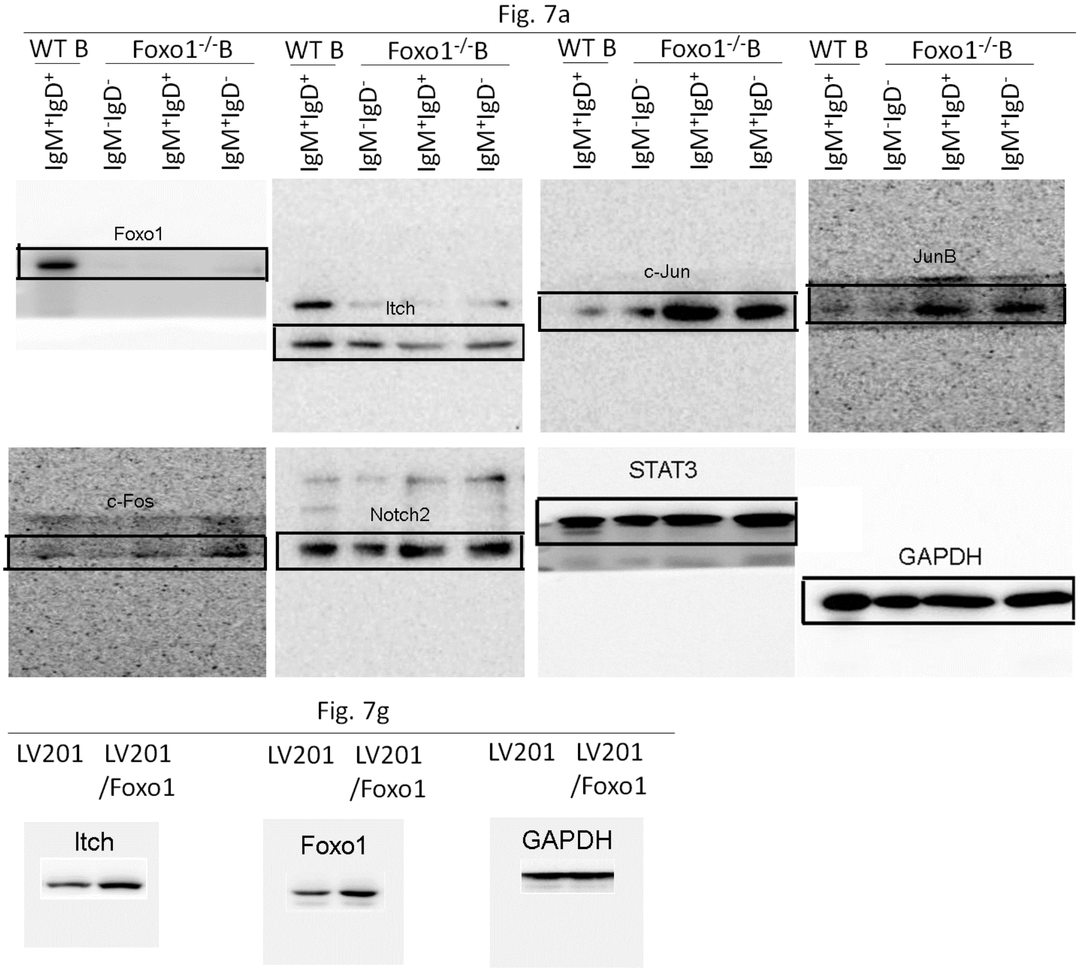

Supplementary Figure 11 original scans for Figure 7a and 7g.

Supplementary Figure 12

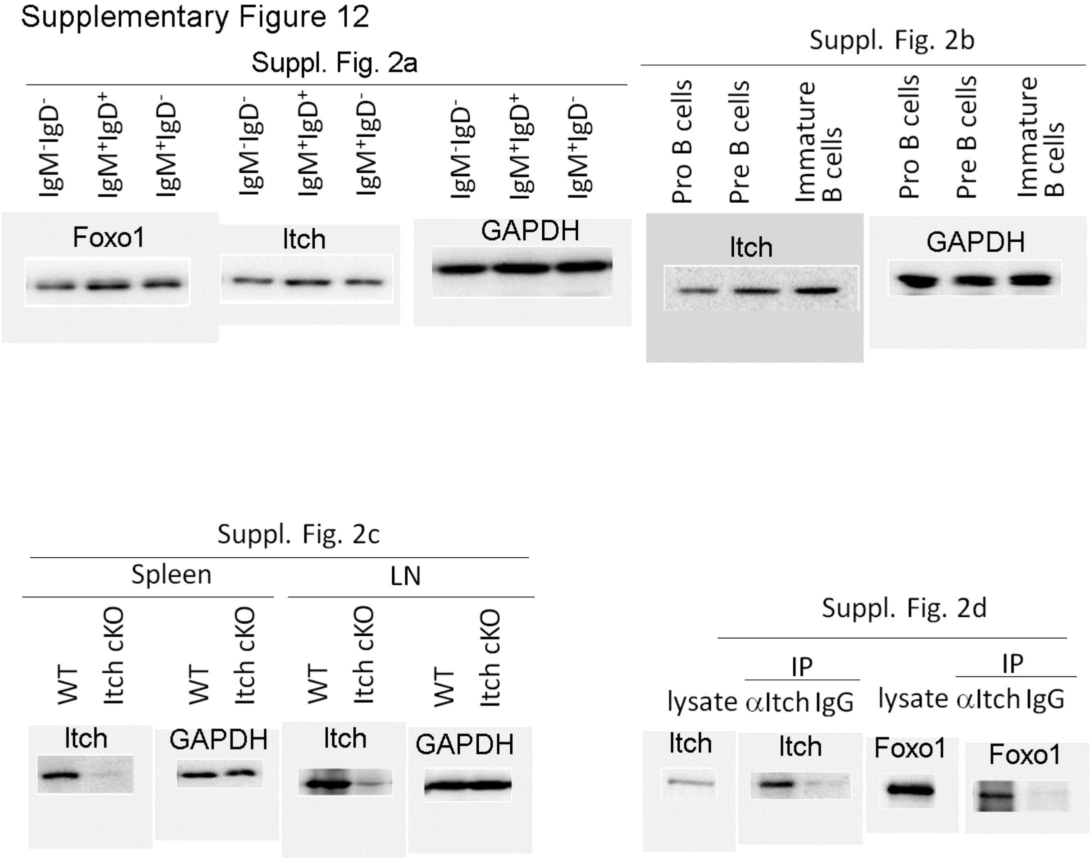

Supplementary Figure 12 original scans for Supplementary Figure 2a-d.

Supplementary Figure 13

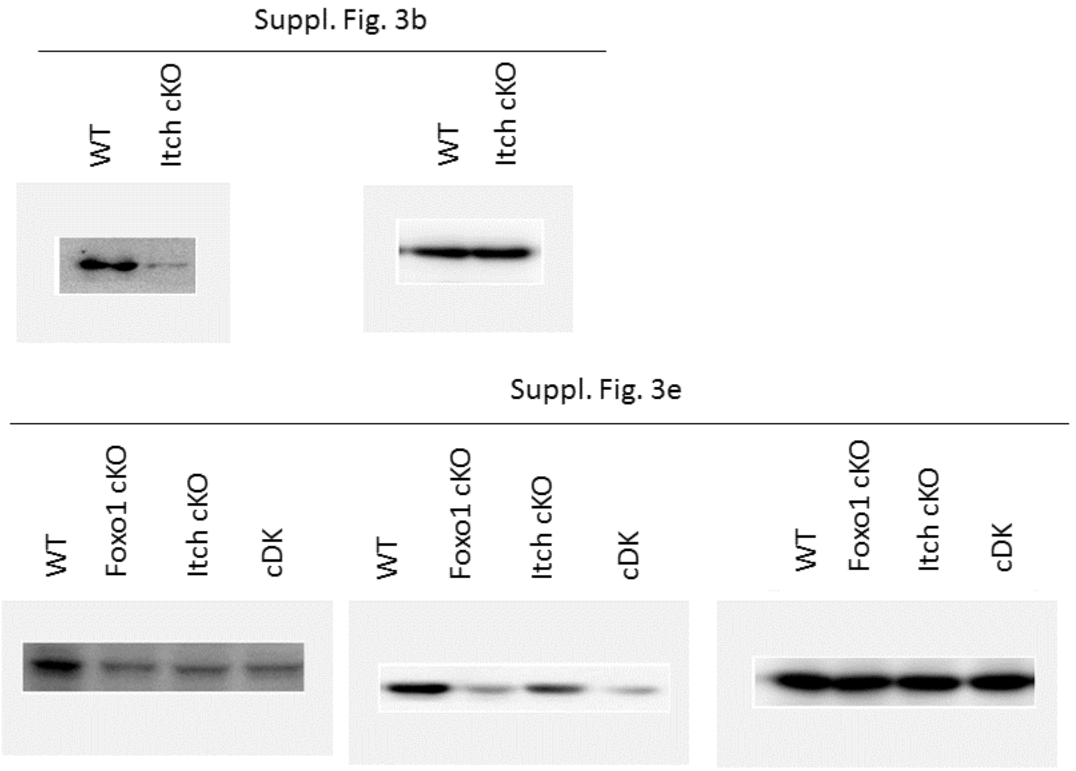

Supplementary Figure 13 original scans for Supplementary Figure 3a and 3e.

Supplementary Figure 14

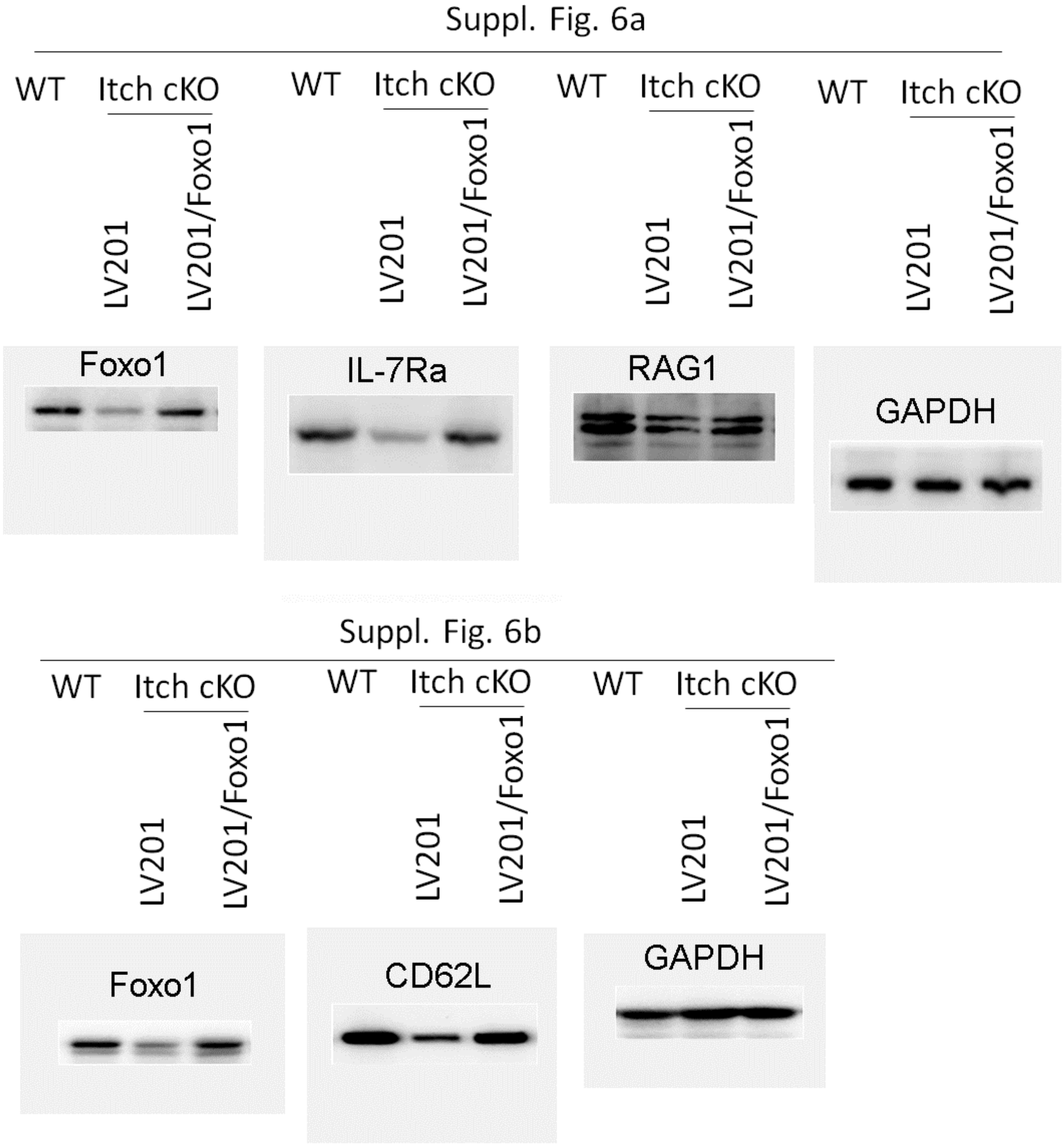

Supplementary Figure 14 original scans for Supplementary Figure 6a and 6b.

Supplemental table I. Mouse Foxo1 promoter sequence.

Mouse Foxo1 promoter sequence was chose from start codon upstream -2465 to downstream +125 of Foxo1 gene:

TTTGTACCAGGCAAAGGCAGGGAACCAGTAAGATGCTCAGTGACGTCCCACGCTTCAGTAAACTGACTTCACACACCACGA
ATAATCACCTCTATGAACCGTTTAAGGTGCATCTGTTATACAAACCTTTGTATTGGGGGCATTGATTGTGCAGATTCTCTGACT
CTTTTCTTTTTTGGTCTTAGCATTGAGCTTAGGGTCCCACTCTTGTTAAGCCTTGGCTGTATCACTAGCCTATACTATGACACTT
ATAGAAACAGTTGGGGACAGTGGCCTTAGAGAATTTGAAGATCTGGGGAAAGCTTTAGGAAAACTCCTTTAACCAAATGCT
CTCATCGCAACAGTCCCTGAGAAACCGAAACAGAAATGAGGCGAAGGCCAAATTCACAGCTGGATTTCATCGCCAAGAATG
GTCCTCTAGTGCTCCGCCTATTTGTAGGGTGCGGCTGTCTGACTGTGGGCCCACCTCGGCAGAGTGTGATTACAAAGGAGCA
CCAGAGGGTTGTTCTTTGTGGTCCTGCTTAGGCTCATCCATTTACTCGAGTCTCCCAGCGGTTGTCAAAGCCAAATTTTGTAG
TCAAGGTGAAGAGACAGTTAACCAAACTGTCCTCGGCACCTCTGGTTCAGGATCGCCACTCAAAACTTTTATCAGTAGGCA
GCCGTTTCAGAAAAAGGGATTATGAATAGGGGAATAGGGGCCTGGATTAGCGAGACGTTAGAGACAAAGTGGAAGGAGTAA
GAAGGCTGGTTTTCTTTTGGAGTCTCATTTTACATAGAAAAACTCCACGTAAGAAAGAAGAGCGAAGTACAACCGCAACAA
GACATTTGGAGCTGCGTTTTTGGACATATTTAAAGCCCTAATATGCATCTTCAATTCTAAAGCATCCTAGCCAATAAAAGAAAA
TGCCACCCATATTTCTTTTTTTTTAAAAAAGAAAGCAAATCAGAGGATCAAGCGAGGGAACATTTTCTTCTATTCTGTAAGGC
AAAAAGAACCACTGTCTTTCTACCTTTGGCTCCGCCGGACGTGTGTTAGATACGCGGGTCTGGGAGCTCACTCTGTGAGTTT
AAAGAACTGAGGGGCGGAGCTGTGCCCCAACACTTGGGGCTTATCTCCTTTTGCGACTTCAACACCTCATCGCTTCCATGCT
AAATAAAAAAGAAATAAGCAAGCAGTTAAACTTAATTAGGGGTAATAAAGTGACAAAGTTGTCTCTAAGCGCTCCTCTGACT
CTTTAGCCACCACACCGGTCCTCAGGAAAAAGTGAGATTCCCGTGGCAGAAAAGCGGTTCCCATAGAAGACGTCGGATCTG
AGGCGCTACTGGAGCTAGGTGCTTGTGCAACAGTGTTCTCCCCGCGCCGCACCTCTCTGGGGTTCAGGATGAGTGGAGGCT
GGGGGCTGGACCGAGCCGCAGAGGCTCGTCTTGCAGGTGTGCACAGGTAGGGTGGGAAGCGGTTACTCCGGAGCCGCAGA
GCGACGGGTCTTCACCGAAGGATCTGCGCGCCTGGGGCGGCCAGGGCAGATGCCAGCTTAAGAAAACATTCCTCCAAAAC
AAACCCCACCGACTTCCCGAGAGCTGCGCGAAGACGCGTGGGCGGAGCGGGCTTGAGTGGAATCCACGAGGCGCCCCACC
GCCCACGTCGCGTCCGGGAGCGCGCAGCGGCCCCGCGCCATCCCGGGAGTCAGGGTCTGGGCTCTGCCTGGAGTACTCGAT
TGCACTCTGTAGTAAACAAAAGTATGGTAGCCGCCTCCACGCCGGTTTGCCTCCTAGCAATCCGATCGCTGAGACGCCAGAG
ACTCAGAAACACTTGAGAATTACAGAAAATAATAACGGAGATTGGAAAAAAAAAAAAAAGTGTGTGAGAAAGAAAAATAC
CCCACCGCCCCCCACCAACCTACTGCCGCCTCCCGAGGGAAGGCAGCGCCCGCCCAGGCCCGCGTTCATTGGCTGCTTCGG
CGGAGACTTCGCGCGTCCATTGGCCGCGCAGCCTGTCAGTCAGGGGCGGGGCGGCGCGCGCCGCCGCGGGCGGGGAGC
CCGCTGCAGATCCCGTAAGACGGGAGTCTGCGGAGTCGCTTCAGTCCCCGCCGCCGCCACATTCAACAGGCAGCAGCGCCG
CTGTCGCGCGGCCGCGGAGAGCTAGAGCGGCCCGCAGCGTCCGCCCGTCTGCCTTGGCGTCCGCGGCCCTTGTCAGCGGGA
GCGCGGTGCCCGAGCTGCCGGGCTCCGCGGCCTGGTCGGTGCCCCGTCCTAGGCACGAACTCGGAGGCTCCTTAGACACCG
GTGACCCAGCGAAGTTAAGTTCTGGGCGCGTCCGTCCGCTGCGCCCCGCCGCGCCTGACTCCGGCGTGCGTCCGCCGTCCG
CGGCCCCCCAATCTCGGAGCGACACTCGGGTCGCCCGCTCCGCGCCCCCGGTGGCCGCGTCTCCCGGTACTTCTCTGCTGGT
GGGGGAGGGGCGGGGGCACCATGGCCGAAGCGCCCCAGGTGGTGGAGACCGACCCGGACTTCGAGCCGCTGCCCCGGCA
GCGCTCCTGTACCTGGCCGCTGCCCAGGCCGGAGTTTAACCAGTCCAACTCGACCACCTCCAGTCC

We used the web-based software (http://www.cbs.dtu.dk/services/Promoter/) to identify potential promoter sequences. As expected, the sequence has obvious characteristics of promoter.

Supplemental table II. Predicted AP1 and JunB binding sites in Foxo1 promoter

Model namer	Score	Relative score	Start	End	Strand	Predicted site sequence
FOS::JUN	8.68	0.92758162	1995	2001	-1	TGACTGA
FOS::JUN	7.614	0.888670875	161	167	1	TGACTCT
FOS::JUN	7.614	0.888670875	1229	1235	1	TGACTCT
FOS::JUN	6.836	0.860272602	2284	2290	1	TGACCCA
FOS::JUN	6.696	0.855162373	55	61	-1	TTACTGA
FOS::JUN	6.656	0.853702308	1681	1687	-1	TGACTCC
FOS::JUN	6.656	0.853702308	2338	2344	1	TGACTCC
FOS::JUN	6.574	0.850709173	1797	1803	1	AGACTCA
JUNB	6.105	0.868819881	2281	2291	1	CGGTGACCCAG
FOS::JUN	5.729	0.819865291	620	626	-1	TGAACCA
FOS::JUN	5.723	0.819646281	243	249	1	TGACACT
FOS::JUN	5.631	0.81628813	452	458	1	TGACTGT
FOS::JUN	5.631	0.81628813	536	542	1	TTACTCG
FOS::JUN	5.549	0.813294996	223	229	1	TGTATCA
JUNB	5.371	0.859328383	1794	1804	1	CAGAGACTCAG
FOS::JUN	5.187	0.800081404	1340	1346	1	TGCAACA
JUNB	5.046	0.855125745	1228	1238	-1	TAAAGAGTCAG
JUNB	4.884	0.853030891	160	170	-1	AAAAGAGTCAG
JUNB	3.69	0.837591043	1992	2002	1	CTGTCAGTCAG
JUNB	2.5	0.822202921	1678	1688	1	CCGGGAGTCAG
JUNB	2.491	0.82208654	2272	2282	-1	CGGTGTCTAAG
JUNB	2.148	0.81765114	222	232	-1	TAGTGATACAG
JUNB	1.996	0.815685598	730	740	1	GAAGGAGTAAG
JUNB	1.856	0.813875231	1199	1209	1	AAGTGACAAAG
JUNB	0.9	0.801513008	1860	1870	1	GTGTGAGAAAG
JUNB	0.854	0.800918173	158	168	1	CTCTGACTCTT
JUNB	0.854	0.800918173	1226	1236	1	CTCTGACTCTT

We used the web (http://jaspar.genereg.net/) to analyze the binding sites of AP1 and JunB in Foxo1 promoter sequence.

Supplemental table III. Mouse miR-182 promoter sequence.

Mouse miR-182 promoter sequence was chose from upstream -3000 of mature miRNA of miR-182 gene:

GTTGTGGCCTTTCCATCCACCCACTAGTGTTGGTTTCCTGCATCCTTGGAGTCCCTGTGACTTAATGTCCCTAAGCTGCCCATA
TATGTGTTATGTAACATTGTGTATGTAAGCTCTGATCTCGAACAGCTGCACCAGTTTGTAAAGAGTCATCTCACAGATGGAGG
GACAGGCTGGAGGTCCATTAGTAGTGAAGTAGGGTCGCTACCTCTCTTCAGTCACGACAATGTATTGACCTCCAGTGGCCTT
GCTCTGTGGAGAGTGCTCACACACCCCTGCCCCCTGTGGCTGGGTGAATCTTGGCTCTGACCCTTCATATATCCCTGGCCTTC
AAAATGAGTTAGATCTGGACCTGGTCCATAGGGCTTAGAAGAGCCCACCCACCAGCCAGGCTCTTGGGTGAAGCCCTGGTG
GTTAGCAGGCAGTGAGAGCTGAGCTGACTAAGCTGGTCCACTGACCATCTGGTAGCCATCATATCTGAAGTTACTGGGCCAG
CCCCTAATGAGACTGCTTCTCCCTGGGGAAGGGGAACTTCTTGCAAGAGCCAGTTACTCAGTCCAGTTACAGTTAGGTGGCC
AAGCTAAAGAGGGCTGGGGCCAGGCCAGGGGAATGTGTACAGTTGAGTCTGGAAGTTAGAGATCAACCAATCCAGTTGACT
GCTCACCAGGAACGTGCCTGAGCATCCAGGGCCGCCCGCCTGCTTGGGTGCCCTGAGAGTTGTGTTGGATAGCGAGGACAT
TTAGACCTACTACTGCCAACTGGGGGCTTCCATAAGTCTCTGGGGTATGGCACTAATGTCCTGTGCAGAAGGCTATCTGAGC
ACTTGCGGGAAAAGGAAGGACAGGAAGAGACCTCACCGCCTCACCCTGGTGAGGTCTCTGTTTCGGGTCTTGCCTCTGTGA
ACCCAACTCTGCTCCTGTCCAGCACTCAGGGGGCCACTCAGAGCTGGGTCCCTCTAACCAAAGCAGTCCTCAGACCCCTAG
TGGTCCTGCCTCTCACACTGGCCTTGGTGGAGGCTTTGCTGAGACCTCACTAGCAGGTGTTGGTATGGCCCAGTTCTATACTT
CTTTCCAGGCTCAGGGCTCCACCCCTCTGGGGCCTGCATTCCCAAAGGATGACCACTGGCCATAGCTTTCTGGTTCTCCTCAT
CTAGCCTGTTCTCGACCTGGGTGAAAGTTCACCTATAGTAGTGACTGCCGGTGAGCAGAGGCCACAAGGCTGGTTGGACTC
CGCAGAAAATCAGCTTCCAGAGGACCATGCTGGTGAGGGTGCGGTAGGAGGGGAGGGCTCACCCTGTCAAGCAGGACCAG
TTTCTTCCTGGCTGTCTGATCCTGCGGAAGGATTTACCCTTACCCTCTCAGGGCAGTTGGTGCAGTTTAATCCACTGGGGACC
TTCTTATATTGTTCGGGCTCGGTGGCTCCTAGGTGCCTGAGTTGCTGCCTCCTGGGTAGGCTCCTTGCCTCATTGCCTGGTGTT
GGGGTTGCCTGGGCCAGCCGCTCCCCAGAGTCCCTGTGAGGTGGATTATTCCAGACTTTCGCCCTTCCCCCACCCCTGCATT
TGCTTATTCAATCCAGGCCATTATCCGGGTCCCTGCCCCAGCTCTGCAGTCCTCTGGCTCTTGGAAATGCGATCTGGCTCCAT
TGATCGCAGGCAGGCGGGGCTGCTCGCTCCCAGCCTAGCTAGTGTGGCCCGAGATTGAGCAGTTAGGGGTGCTGACAGGAT
AGGACCCTCCCCGCTCACACTGGAGTGGAGGGAGTGACCCACTCAACTCACCAGTGGCTTGCCTTAACACTTTATAATGTGA
TAAAAATGTGTGGCCTGCCTTTGGGAGGTATGTGGCCAAGAAACCAAGGTCCTGCTTGTGTCCCCACTGGGGACACACTCT
AGGTCAAGGTTTCAAGGAGCTTCCTTGCCAAGCAGATCACCCCAGCCCCTGAACTCAGTACTGGGATCACAGATGTGGCCC
ACAATTCTGGCCCAGCTATAAAGCAAGTTTTTCTCTGGGCAGTGGAGTCATCTCAGCACCCAAGCTTAGGCCACTGGCAGTG
ACCAGCTTGAGTGTCCAGGGTTCGTCTGCATCTCTGCCCTTCCAAAACATCATCTTTGGGTCCTTCATCCTCCCTGCCCTGCA
GGAGGACAGAGGGACCGGTGGCATTTCCATCACTGGCCCTGCCAGCCAGCAGTTCCTCTAGATTTATGGAAATGAATCCATG
GGTGGTTTGGGGGCAAAGAAGTGTACCATCTTTGGGGTTCCTTGAAGGCTGCAGGGGTGTGTGTCTTTTTGGAAGGGATGG
GTAAGGAAATCTCTGCACTTGGGAGAATGCTGTGTGGATGTGCTAATGCAGCTGTGTTCCGCTTGGCTGGCCCTGCTAGACT
GCAGTGATGGCTGTCTAGACTCTCCAGAGAAGGTCTTAGCCTAGGGTTCTTCCTCCCCAGCCTCAGGGAGGAGACCACAGG
CCGGGCAGTGGTTTGCCTGTGGCTTGACAAGGAAGTGGGATCTATGCATTGACCTTTTACCTCCCTCCCGGTGGGGGGGTGG
GACAGCTGAGGAACTTTAGGCTCTGGGGAATGTCAGGAAGGGATTAAACAGGATGCCACCTGGGGGCCTTGGTAGATGGCA
AGCTGGCCCCATGCCACTCTCTTCCTTGGTTACCAAGCAGGGGACTCCCTGACCCAGAGTGCACCCATTCCCAAGCTCCCAC
TGGCAGGTTCCTGGGGTACAGTGCCTTTGAGAGCAGTGGCTATGGCTCCATCACTGCCTAATGCCCCTACACAGGGACTGTG
GGTCGTGGGGAGGGGGGGAGGTGGTCGTGGGGTGGGGGGGAGGTGGCTGGAGGGCAGGCAGGGCTTGAGGAGGTTTTAC
ACTGGAACAGGACCATACAGGCCGAAGGACCATAGTCTGGACCTTGTGTTAACTGTGGGAAGAGCGCCCTCCTAAAACCAC
CCTAACTGCTTCTTCTTCAGCATAGGCTTACTGGTCTGGCTGCTGGAGGCCTCCCACCATTTTTGGCAATGGTAGAACTCACA
CCGGTAAGGTAATGGGACCCGGTGGTTCTAGACTTGCCAACTATGGT

We used the web-based software (http://www.cbs.dtu.dk/services/Promoter/) to identify potential promoter sequences. As expected, the sequence has obvious characteristics of promoter.

Supplemental table IV. Predicted JunB binding sites in miR-182 promoter

Model name	Score	Relative score	Start	End	Strand	predicted site sequence
JUNB	1.448	0.808599	55	65	1	CTGTGACTTAA
JUNB	5.276	0.8581	143	153	1	TAAAGAGTCAT
JUNB	6.495	0.873863	145	155	-1	AGATGACTCTT
JUNB	4.994	0.854453	334	344	1	AAATGAGTTAG
JUNB	0.952	0.802185	336	346	-1	ATCTAACTCAT
JUNB	7.906	0.892109	435	445	1	AGCTGACTAAG
JUNB	1.842	0.813694	437	447	-1	AGCTTAGTCAG
JUNB	5.371	0.859328	546	556	1	CAGTTACTCAG
JUNB	6.212	0.870204	548	558	-1	GACTGAGTAAC
JUNB	1.786	0.81297	1458	1468	-1	CAATGAGGCAA
JUNB	5.157	0.856561	1755	1765	1	GAGTGACCCAC
JUNB	4.509	0.848182	1757	1767	-1	GAGTGGGTCAC
JUNB	3.265	0.832095	2010	2020	-1	AGATGACTCCA
JUNB	2.73	0.825177	2290	2300	1	GGATGGGTAAG

We used the web (http://jaspar.genereg.net/) to analyze the binding sites of JunB in miR-182 promoter sequence.

Supplemental table V. Predicted binding of both 3’-UTR of Foxo1 and miR-182.

```
2701 5’ATCACTTAAAATGGAAACAGTTCCCCAAAGGTGTTCAATTTGAACTTGTTCAACTGCTTA 3’  Foxo1 3’-UTR
           ||||  ||  ||||  |||  |||
1     3’-ACACTCAAGATGGTAACGGTTT -5’                                  miR-182
```

Supplemental table VI. Mouse Itch promoter sequence.

Mouse Itch promoter sequence was chose from start codon upstream -1938 to downstream +100 of Itch gene:

AAATGCAAAACCCATGTAGGCAAGCTGAGTGTGGTAATTCATGCTTGTAATCTCATTACTGGGAAGGTTGAGCGTGAAGCAT
CCTGAGCATGAGGCCAGTCTGAGTTAGTTAGTGAGAATTTGTCCCCTAAATAAAGAAAAAACTGTAGATAAAGATTATCTGG
AACTAGATGACTTGGCTTGGAACCTTGGTTTCTGTGTTAGTGATCTTGGGCCAATTAATAATTTCCTTTATATTCAGTTGTTTTC
TGTACAAAATAATAAAGTAACAAAATATATTTATAATATTTGTTAAAATATCTTATAGTATGCCCAGTGACAAGAATCATCTGGG
ATGCTAGTTTAAAAAACAAAACAAAACGAAACAAGAAACCAACCCAGATTCTCCTAGAGCACACCACTCTCTCAATATTGG
GGATAAAATCTTGGGCCTCATCAATGCTATGTAAGTATCTACTAGTGAGTTACATCTTGAGCCAACTCTTACAGCTTAATCCCT
AATGTGGCAAACATTGGCTGGAAGTGCAGAAGAACTTTTAAATACTTAAACACACCAAACCAAAACTAAAAAACAAAAAC
AAAATCCCAAAAACCCAAACCCAGGGTTTCACCACGTAACCTCGGTTGGTCTAGAACTCTCAAGATATTTGGACATGGCTAG
CCTTGAACCCACAGAGATCCATTTGTCTCTGTCTTCTAATGGAACCTGAAGCTTGCTGTGTGGTCTAGGGCGTTGACTAGGG
AGCTCCTATGTCTGCCCACCACTGTTAGGGGTCCGTTGGTGCCAGCTTTTATATGAATGCTGGGGTTTCAAACATCCTTGTGC
TTGCATGGCAAGAGCCTTTACCCACTGAACCATCCTCCCAACTCAGAATCTTTTTTGTTTAATAAACTCAGCACATAATTCATA
ATATGGTAGTTTCAAAGGTATTTCGAAAAGTTTAAGTGACTTTCTAGATAGTTTCCTTACGAGGAAACAGGATAAACAATAAT
AGCAAAGTTTGCTAATTATCACGTGCCTCTATTATTACCACTGCACAGCCTATTAATTCGGTAGGATTAGATGTGTTTTGCCTG
GGTTAAGAAGTTTAATTTAAGCATCTGAAAAGGTAACCAATGGCATCTTTCACATAGTTTTGTTGTAAACGTTTAGAAGTATG
CTTTTCAGTTTTTATAACTCATGCCGCCTTGGCGCTGTCAAGCTGTCACCCTCCTAAGAACTTCCTGTCAAAAGGTGGAGAA
AGGAGAATTTGAACCCAAACAGTGTAACTCCAGAAGGATAGAACCGGCACAGTGCTTCATAAATTGTGATTCCTGCTGTTAG
GAGTATCAGGAGAGAACTTGATAAAAATGCAGATTATAAAGCAACCCCTATTCCATCCCAGATCTACTGAGTCAGAAACTGG
CGGTGGAGCTCAGCAGTCTGTTTTTAAAAGCTGTTCAAGTGATTCTGAGACACGCTAGCATTTGGTAACAGTCTTTAGAGGT
CCTGGCGGGGAAGAAGGGGTCAAAAGGAAGGAACGAAAGAAGAAAGGAAAGAAGGGAGAAAGGAAAGGAGAAAGGAA
GGAAGAAAAGAAAAAAGCCTTGGAGATGTAGAGAGCAAGGCTTAGTGTCCACTTCACCTGGAGGTGAGGAAAAATGATTT
AGGAGGCTGGCGCGCAAGGCCTGCTGGGGCCTGTAGTCCGGACTGCGCCGAGGAGGCCGCGCCTGCGCACTAGGTGTGAA
GTAGCTCTAGTCGCTGTAGTCGGGGCTCGGGACCGGCTGCCATCTTAGCGGAGGGACTGAGGAGTCGTCGCCGCCCCGAGT
CCCGGTAACATGCATTTCACAGTGGCCTTCTGGAAACAACGCCTTAACCCTAAGAAGTGACTCAGCCATTGAGAACTTCAGG
TTTGCTGTCTTATTGATGGTATGTCTGACAGTGGACCACAGCTTGATTCCATGGGTAGTCTGACCATGAAATCTCAACTTCAG
ATCACTGTCATCTCAGCCAAACTTAAAGAAAATAAAAAGAATTGGTTTGGACCAAGTCCTTATGTAG

We used the web-based software (http://www.cbs.dtu.dk/services/Promoter/) to identify potential promoter sequences. As expected, the sequence has obvious characteristics of promoter.

Supplemental table VII. Predicted Foxo1 binding sites in Itch promoter

Model name	Score	Relative score	Start	End	Strand	predicted site sequence
Foxo1	4.051	0.803590151	131	141	-1	TTTTCTTTATT
Foxo1	3.852	0.800553398	187	197	1	CCTTGGTTTCT
Foxo1	11.474	0.916865639	240	250	1	AGTTGTTTTCT
Foxo1	9.71	0.889946878	345	355	-1	TTTTGTTTTTT
Foxo1	4.399	0.808900655	350	360	-1	TTTTGTTTTGT
Foxo1	4.82	0.815325144	360	370	-1	TCTTGTTTCGT
Foxo1	3.856	0.800614438	368	378	-1	GGTTGGTTTCT
Foxo1	9.71	0.889946878	567	577	-1	TTTTGTTTTTT
Foxo1	6.474	0.840565297	573	583	-1	TTTTGTTTTTG
Foxo1	6.388	0.839252931	879	889	1	TTTTGTTTAAT
Foxo1	12.984	0.939908342	972	982	-1	TCCTGTTTCCT
Foxo1	9.969	0.893899236	981	991	-1	TATTGTTTATC
Foxo1	4.974	0.817675194	1226	1236	-1	ACCTTTTGACA
Foxo1	9.616	0.888512432	1422	1432	1	GTCTGTTTTTA
Foxo1	5.497	0.82565621	1506	1516	-1	TCCTTTTGACC
Foxo1	4.089	0.804170034	1569	1579	-1	TTTTCTTTTCT
Foxo1	4.681	0.813203994	1574	1584	-1	GGCTTTTTTCT
Foxo1	11.97	0.924434632	1810	1820	-1	GCGTGTTTACC

We used the web (http://jaspar.genereg.net/) to analyze the binding sites of Foxo1 in Itch promoter sequence.

Supplemental table VIII. List of anti-mouse antibodies used for flow cytometry (FC) and immunoblot (WB) analysis.

Molecule	Application	Species	Fluorochrome	Clone	Manufacturer
Anti-mouse antibodies					
B220	FC	Mouse	PerCP-Cy5.5	RA3-6B2	eBioscience
B220	FC	Mouse	PerCP	RA3-6B2	eBioscience
B220	FC	Mouse	APC	RA3-6B2	eBioscience
B220	FC	Mouse	FITC	RA3-6B2	BioLegend
CD19	FC	Mouse	FITC	6D5	BioLegend
CD19	FC	Mouse	APC	6D5	BioLegend
IgM	FC	Mouse	FITC	eB121-15F9	eBioscience
IgM	FC	Mouse	PE	11/41	eBioscience
IgD	FC	Mouse	PerCP	11-26c.2a	BioLegend
IgD	FC	Mouse	PE	11-26c	eBioscience
IgD	FC	Mouse	FITC	11-26c	eBioscience
CD43	FC	Mouse	APC	S11	BioLegend
CD3	FC	Mouse	FITC	17A2	BioLegend
CD3	FC	Mouse	PerCP	145-2C11	BioLegend
CD62L	FC	Mouse	FITC	MEL-14	BioLegend
IL-7Ra	FC	Mouse	FITC	A7R34	eBioscience
IL-7Ra	FC	Mouse	PE-Cy7	A7R34	eBioscience
CD93	FC	Mouse	FITC	AA4.1	eBioscience
μ	FC	Mouse	APC	II/41	eBioscience
κ	FC	Mouse	APC	187.1	eBioscience
λ	FC	Mouse	APC	R26–46	eBioscience
CD8a	FC	Mouse	PE	53-6.7	BioLegend
CD8a	FC	Mouse	FITC	53-6.7	BioLegend
Foxo1	WB	Rabbit	Purified	C29H4	Cell Signaling Technology
Itch	WB	Rabbit	Purified	D8Q6D	Cell Signaling Technology
GAPDH	WB	Rabbit	Purified	FL-335	Santa Cruz Biotechnology
IgG	WB	Rabbit	Purified	D2814	Santa Cruz Biotechnology
RAG1	WB	Rabbit	Purified	H-300	Santa Cruz Biotechnology
IL-7Ra	WB	Rabbit	Purified	EPR2955(2)	Abcam
CD62L	WB	Rabbit	Purified	Ab18959	Abcam
Ub	WB	Mouse	Purified	AB19247	Abcam
Ub	WB	Rabbit	Purified	P4D1	Santa Cruz Biotechnology
JunB	WB	Rabbit	Purified	C37F9	Cell Signaling Technology
c-Fos	WB	Rabbit	Purified	9F6	Cell Signaling Technology
p-c-Jun	WB	Rabbit	Purified	54B3	Cell Signaling Technology
c-Jun	WB	Rabbit	Purified	60A8	Cell Signaling Technology
Notch2	WB	Rabbit	Purified	D76A6	Cell Signaling Technology
STAT3	WB	Rabbit	Purified	79D7	Cell Signaling Technology

Secondary Reagents					
IgG (H+L)	WB	Rabbit	HRP		GE Healthcare
IgG (H+L)	WB	Mouse	HRP		GE Healthcare

Supplemental table IX. List of primers for Chip-qPCR, miRNA-qPCR, Real-time quantitative PCR and PCR.

All primers for Mus musculus	Forward primer	Reverse primer	Product size
ChIP-qPCR primer pairs			
Foxo1 promoter primer pair p1	5’-GTCCCACGCTTCAGTAAACT-3’	5’-TTCTCTAAGGCCACTGTCCC-3’	239 bp
Foxo1 promoter primer pair p2	5’-AAGATGCTCAGTGACGTCCC-3’	5’-GAGTGGGACCCTAAGCTCAA-3’	178 bp
Foxo1 promoter primer pair p3	5’-GCTGGATTTCATCGCCAAGAA-3’	5’-CTGGGAGACTCGAGTAAATGGA-3’	163 bp
Foxo1 promoter primer pair p4	5’-TGTCTCTAAGCGCTCCTCTG-3’	5’-CTCCACTCATCCTGAACCCC-3’	182 bp
Foxo1 promoter primer pair p5	5’-ACAAAAGTATGGTAGCCGCC-3’	5’-GAGGCGGCAGTAGGTTGG-3’	179 bp
miR-21 promoter primer pair	5'-CCTTTCCCACCCTCAAAAAG-3'	5'-TGGCGTTGGTGACTTAGTTG-3'	126 bp
miR-155 promoter primer pair	5'-GGTTCCCCTCTGGTCCTAAA-3'	5'-GGGACTTGTCTCCAGCAGAG-3'	195 bp
miR-182 promoter primer pair P1	5'-CTCACAGATGGAGGGACAGG-3'	5'-CATTTTGAAGGCCAGGGATA-3'	185 bp
miR-182 promoter primer pair P2	5'-GGTTAGCAGGCAGTGAGAGC-3'	5'-TGGCCACCTAACTGTAACTGG-3'	166 bp
Itch promoter primer pair p1	5'-GCTTGGAACCTTGGTTTCTG-3'	5'-GCATCCCAGATGATTCTTGTC-3'	168 bp
Itch promoter primer pair p2	5'-CATTGATGAGGCCCAAGATT-3'	5'-ATGCCCAGTGACAAGAATCA-3'	133 bp
Itch promoter primer pair p3	5'-GGATCTCTGTGGGTTCAAGG-3'	5'-GGCTGGAAGTGCAGAAGAAC-3'	167 bp
Itch promoter primer pair p4	5'-GCTTGCTGTGTGGTCTAGGG-3'	5'-TTCTGAGTTGGGAGGATGGT-3'	163 bp
Itch promoter primer pair p5	5'-TGCCATTGGTTACCTTTTCA-3'	5'-TTTCCTTACGAGGAAACAGGA-3'	161 bp
Itch promoter primer pair p6	5'-AAGCAACCCCTATTCCATCC-3'	5'-TCCTTCCTTTTGACCCCTTC-3'	158 bp
Itch promoter primer pair p7	5'-TGCCTTTTCATCCAAACTCC-3'	5'-CTGCGCACTAGGTGTGAAGT-3'	166 bp
miRNA-qPCR primer pairs			
miR-21 reverse transcription primer		5'-GTCGTATCCAGTGCAGGGTCCGAGGT-ATTCGCACTGGATACGACTCAACATC-3'	
miR-21 qPCR primer pair	5'-GCGCTGGAATGTAAAGAAGTttaatgctaattgtga-3'	5'-GTGCAGGGTCCGAGGT-3'	78 bp
miR-155 reverse transcription primer		3'GTCGTATCCAGTGCAGGGTCCGAGGT-ATTCGCACTGGATACGACCCCCTATC-3'	
miR-155 qPCR primer pair	5'-GCGCTGGAATGTAAAGAAGTttaatgctaattgtga-3'	5'-GTGCAGGGTCCGAGGT-3'	78 bp
miR-182 reverse transcription primer		5'-GTCGTATCCAGTGCAGGGTCCGAGGT-ATTCGCACTGGATACGACTGTGAGTT-3'	
miR-182 qPCR primer pair	5'-GCGCTGGAATGTAAAGAAGTtttggcaatggtagaa-3'	5'-GTGCAGGGTCCGAGGT-3'	78 bp
U6	5'-CTCGCTTCGGCAGCACA-3'	5'-AACGCTTCACGAATTTGCGT-3'	78 bp
Real-time quantitative PCR			
Foxo1 primer pair	5'-TTCAATTCGCCACAATCTGTC-3'	5'-GGGTGATTTTCCGCTCTTGC-3'	118 bp
PCR for Genotype			
Itch floxed gene	5'-ACAAGAGGTAGGAGACAAGCATT-3'	5'-TGCAGCTTATTTATCATTCCTTA-3'	wt 237 bp flox/flox 270 bp
Foxo1 floxed gene	5'-GCTTAGAGCAGAGATGTTCTCACATT	5'-CCAGAGTCTTTGTATCAGGCAAATAA-3'	WT 115bp flox/flox 149 bp
CD19 cre gene	5'-ATTTGCCTGCATTACCGGTC-3'	5'-ATCAACGTTTTCTTTTCGG-3'	350 bp
CD19 WT	5'-CCTCTCCCTGTCTCCTTCCT-3'	5'-TGGTCTGAGACATTGACAATCA-3'	490 bp

Supplemental table X. Mouse miRNA mimics and inhibitors.

MircoRNA Negative control (N.C)	Sense 5'-UUCUCCGAACGUGUCACGUTT-3'
	Antisense 5'-ACGUGACACGUUCGGAGAATT-3'
MMU-miR-21 mimics	Sense 5'-UAGCUUAUCAGACUGAUGUUGA-3'
	Antisense 5'-AACAUCAGUCUGAUAAGCUAUU-3'
MMU-miR-155 mimics	Sense 5'-UUAAUGCUAAUUGUGAUAGGGGU-3'
	Antisense 5'-CCCUAUCACAAUUAGCAUUAAUU-3'
MMU-miR-182 mimics	Sense 5'-UUUGGCAAUGGUAGAACUCACACCG-3'
	Antisense 5'-GUGUGAGUUCUACCAUUGCCAAAUU-3'
MircoRNA inhibitor N.C	5'-CAGUACUUUUGUGUAGUACAA-3'
MMU-miR-21 inhibitor	5'-UCAACAUCAGUCUGAUAAGCUA-3'
MMU-miR-155 inhibitor	5'-ACCCCUAUCACAAUUAGCAUUAA-3'
MMU-miR-182 inhibitor	5'-CGGUGUGAGUUCUACCAUUGCCAAA-3'
